# Bidirectional transcription of a novel chimeric gene mapping to mouse chromosome Yq

**DOI:** 10.1186/1471-2148-7-171

**Published:** 2007-09-24

**Authors:** Peter JI Ellis, Lydia Ferguson, Emily J Clemente, Nabeel A Affara

**Affiliations:** 1 Mammalian Molecular Genetics Group, University of Cambridge Department of Pathology, Tennis Court Rd., Cambridge, CB2 1QP, UK

## Abstract

**Background:**

The male-specific region of the mouse Y chromosome long arm (MSYq) contains three known highly multi-copy X-Y homologous gene families, *Ssty1/2*, *Sly *and *Asty*. Deletions on MSYq lead to teratozoospermia and subfertility or infertility, with a sex ratio skew in the offspring of subfertile MSYqdel males

**Results:**

We report the highly unusual genomic structure of a novel MSYq locus, *Orly*, and a diverse set of spermatid-specific transcripts arising from copies of this locus. *Orly *is composed of partial copies of *Ssty1*, *Asty *and *Sly *arranged in sequence. The *Ssty1- *and *Sly-*derived segments are in antisense orientation relative to each other, leading to bi-directional transcription of *Orly*. Genome search and phylogenetic tree analysis is used to determine the order of events in mouse Yq evolution. We find that *Orly *is the most recent gene to arise on Yq, and that subsequently there was massive expansion in copy number of all Yq-linked genes.

**Conclusion:**

*Orly *has an unprecedented chimeric structure, and generates both "forward" (*Orly*) and "reverse" (*Orlyos*) transcripts arising from the promoters at each end of the locus. The region of overlap of known *Orly *and *Orlyos *transcripts is homologous to *Sly *intron 2. We propose that *Orly *may be involved in an intragenomic conflict between mouse X and Y chromosomes, and that this process underlies the massive expansion in copy number of the genes on MSYq and their X homologues.

## Background

The mammalian Y chromosome is constitutively haploid, restricted to males, and subject to ongoing genetic deterioration due to lack of recombinational exchange with a homologous partner. Set against this, however, there is strong evolutionary drive to preserve the function of male-benefit genes on the Y chromosome, and to acquire novel male-benefit genes on the Y [1-7]. These opposing effects lead to a heterogeneous structure of Y chromosomal DNA, with functional genes (often male specific, sometimes highly amplified) set among a sea of degenerate pseudogenes, repetitive sequence, and parasitic transposable elements.

The long arm of the mouse Y chromosome is a spectacular example of this process, being highly repetitive, transcriptionally silent in the majority of cell types, and yet indispensable for normal spermatogenesis [8-14]. Deletions on mouse Yq lead to teratozoospermia and reduced fertility. The severity of the phenotypes varies according to the extent of the deletion, with large deletions (> = 9/10 of Yq) resulting in complete infertility [8,13], while smaller deletions (~2/3 of Yq) result in reduced fertility and a less severe sperm shape abnormality [9,11,14]. Intriguingly, the offspring of males with 2/3 Yq deletions show an approximately 60:40 sex ratio skew in favour of females [9], and this is due to reduced efficiency of Y-bearing sperm [15].

Recently, we have made considerable progress in defining the gene content of mouse Yq, identifying two new repeat gene families (*Sly, Asty*)[16] in addition to the one family previously known (*Ssty1*/2). During this work, we observed novel "recombinant" transcripts arising from loci that contain exons from both *Ssty1 *and *Asty*, and termed this new transcript *Asty(rec) *[16]. Here, we describe the detailed genomic arrangement of this rearranged locus and show expression of a large variety of transcriptional variants arising from these rearranged loci. These variant transcripts are differentially regulated during testis development.

We were also interested to know how these rearranged loci arose, and whether there were further examples of such "exon shuffling" on mouse Yq. We therefore compared the genomic organisation of the loci encoding all known Yq genes to each other and to their X-linked homologues, in order to more clearly delineate the composition of the novel rearranged loci, the differences between each of the Yq genes and their X-linked relatives, and the sequence of events involved in the genesis and amplification of these genes.

Finally, we investigated the wider genomic context of the rearranged loci by *in silico *mapping of the location of all known MSYq genes within the currently-released draft Y chromosome sequence contigs. The MSYq gene copies located by the mapping project were used to construct phylogenetic trees elucidating the sequence of events in MSYq evolution

## Results

### A rearranged locus formed by chimerism between three Yq-specific genes

**Figure 1:**
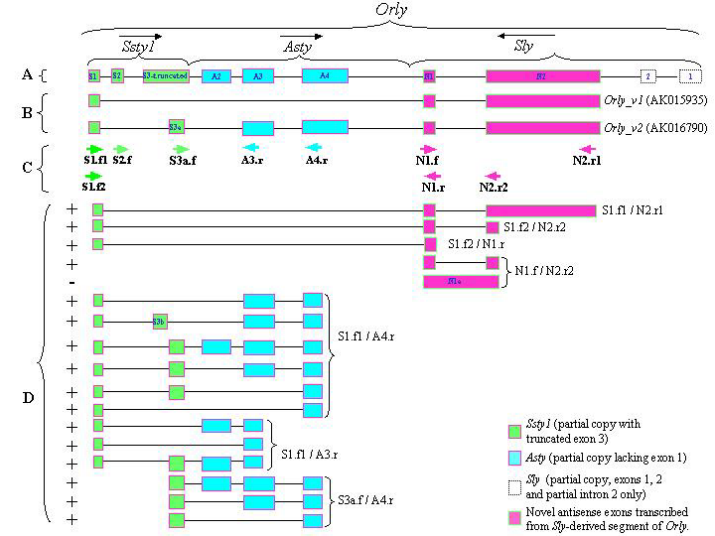
Characterisation of *Orly *transcripts. **A**: Structure of *Orly *as a chimera of relict partial *Ssty1*, *Asty *and *Sly *loci (not to scale). Arrows indicate orientation of the relict loci. Exons are coloured according to their origin. **B**: Structure of *Orly *transcripts found in the nr database. **C**: Primer locations used to characterise *Orly *splice variants (sequences given in **Table 1**) **D**: Structure of variant transcripts detected in the screen. The primer pairs which amplify each variant form are indicated. Exons 3a and 3b are short forms of *Ssty1-*derived exon S3, and are variably included in *Orly *transcripts. Of note is that the majority of splice variant forms do not conform to the splicing patterns of the known transcripts shown in panel **A**. + indicates "forward" transcripts - indicates the "reverse" transcript detected with primer pair N1.f/N2.r2

While we previously reported the presence of both *Ssty1 *and *Asty *exons within the locus encoding *Asty(rec) *[16], further analysis of public genome sequence data shows this locus to be entirely composed of regions with high sequence similarity (greater than 90% across all regions) to members of all three known Yq-linked gene families. The structure of this novel locus is shown in Figure 1A. It comprises sequence homologous to exons 1, 2 and partial exon 3 of *Ssty1*, followed by exons 2–4 of *Asty*, followed by exons 1–2 (and partial intron 2) of *Sly *in the opposite orientation. Since the locus contains segments of all three Yq-linked gene families, we feel the designation *Asty(rec) *is no longer appropriate. Given its highly unusual composition, and the fact that it is transcribed in both directions, we propose the name *Orly *(Oppositely-transcribed, reassorted locus on the Y).

As previously detailed [16], a search of the nr database revealed two full-length cDNAs arising from *Orly*, both originating from the relict *Ssty1 *promoter. In this article we will refer to these transcripts as *Orly_v1 *(accession number [GenBank:AK015935]) and *Orly_v2 *([Genbank:AK016790], referred to in our previous work as *Asty(rec)*). The splicing patterns of *Orly_v1 *and *Orly_*v2 are shown in Figure 1B.

### Orly generates a wide diversity of alternative splice variants

**Figure 2:**
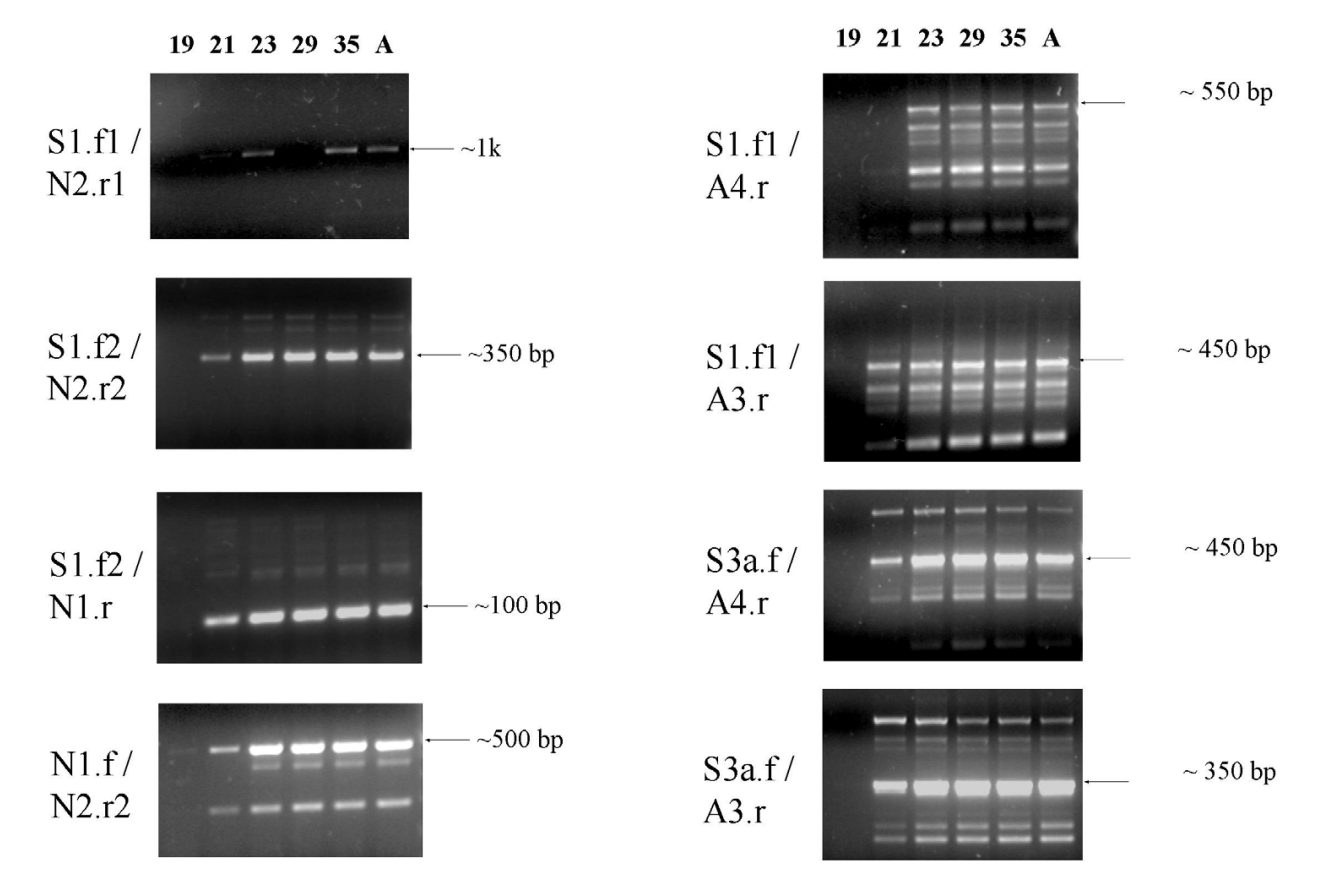
RT-PCR reactions demonstrating the wide range of *Orly *splice variants expressed in developing testis. RT-PCRs were performed on RNA from testes at 19, 21, 23, 29 and 35 days post partum, and on adult (8 week) testes. Size is indicated for one band in each case.

**Figure 3:**
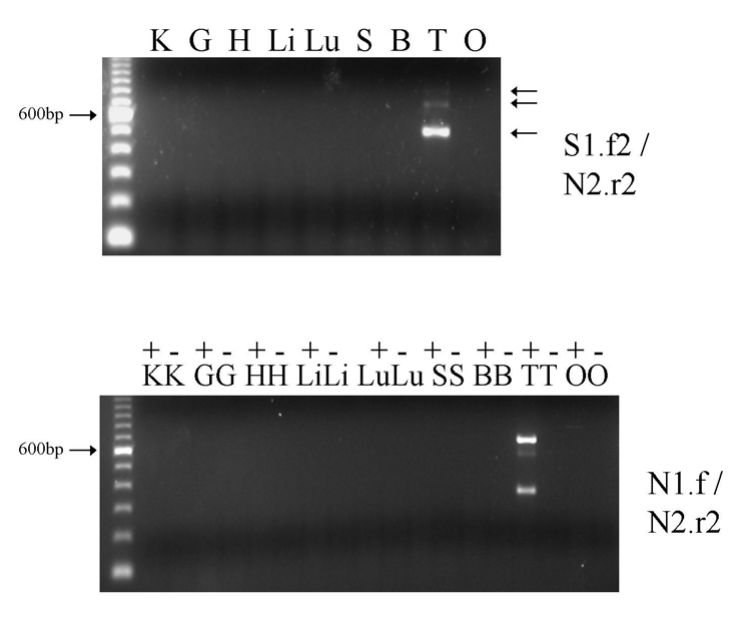
RT-PCR using primer pairs S1.f2/N2.r2 and N1.f/N2.r2 across a range of different tissues. K = kidney, G = gut, H = heart, Li = liver, Lu = lung, S = spleen, B = brain, T = testis, O = ovary. For the N1.f/N2.r2 primer pair, reactions were performed with (+) and without (-) reverse transcriptase. The absence of a band in the – controls demonstrates that the larger unspliced products are true transcripts and not due to DNA contamination.

The existence of the *Orly_v1 *and *Orly_v2 *transcripts indicates differential splicing of the central exons in *Orly*. We performed a screen using primers from the various exons present in *Orly_v2 *(locations indicated in Figure 1C, sequences in Table 1) in order to see what further *Orly *splice variants were expressed. All primer pairs that gave an RT-PCR product from adult testis were tested using a range of normal mouse testis RNAs from different ages *post partum *in order to resolve the developmental onset of expression for each splice variant form (Figure 2). Primer pairs S1.f2/N2.r2 and N1.f/N2.r2 were also tested against a range of tissues to check tissue specificity of expression, and proved to be testis specific (Figure 3).

**Table 1:** Primers used to characterise *Orly *transcripts (locations shown in Figure 1C)

**Primer**	**Sequence**
**S1.f1**	CCCAACGTTGTTCCTCAAGT
**S1.f2**	TCCCAGTGGTGTATGAAAGG
**S2.f**	CGGAGTTGCAGTGGCAAATA
**S3a.f**	AGCTCCTGGATGACTACAAG
**A3.r**	GCAACTCCTCATCAGAGACT
**A4.r**	CGATGATGAGTGACCTAAAGAT
**N1.f**	CAAGATTTGGCATCTGTAAGG
**N1.r**	GCCATTGTCTGATGAAAGTACC
**N2.r2**	CCAGAAGCGGTTACATGC
**N2.r1**	AGCTACCTGAGGAATTGAGAT

When using primer pairs directed at the outermost exons (S1 and N2), a single major band corresponding to *Orly_v1 *is observed, suggesting that this is the most abundant *Orly *transcript. Two faint larger bands were also detected by the S1.f2/N2.r2 primer pair (arrowed in Figure 3), however, we were unable to obtain sequence for these products. These upper bands are likely to represent transcripts including portions of *Ssty1 *exons 2/3 or *Asty *exons 2–4, as detected in the other reactions (see below). No larger bands were seen in the S1.f1/N2.r1 reaction. It is possible that the larger bands detected by the S1.f2/N2.r2 primer pair arises from copies of the *Orly *locus where the exon S1.f1 and/or N2.r1 primer binding sites are mutated.

Using primer pairs directed at the other *Orly_v2 *exons gave a wide variety of bands. Many of the products generated from RT-PCR on adult testis were gel purified and sequenced to confirm which regions of *Orly *are included in each detected transcript. Unfortunately we were unable to generate clean sequence for the products produced by the S3a.f/A3.r primer pair. This is likely due to the presence of a large number of similarly-sized transcripts which cannot be separated on a gel. The resulting partial transcriptional map for *Orly *is shown in Figure 1D. In most cases, the sequenced bands correspond to spliced transcripts, however, the majority of the products do not conform to the splicing pattern of *Orly_v1 *or *Orly_v2*. It appears that there is a plethora of different *Orly *isoforms expressed at low levels, which are only detected when specific primers are used.

We investigated whether any of the detected *Orly *isoforms had any significant coding potential. The *Ssty1 *open reading frame is encoded by exon 3 of *Ssty1*, which is not fully included in any *Orly *transcriptional variant (though two shorter forms of this exon are variably included). *Orly *transcripts thus do not encode the SSTY1 protein. The *Sly *portion of the locus is in antisense, thus *Orly *transcripts cannot encode any portion of SLY. Finally, *Asty *does not contain any open reading frame, thus the *Asty*-related portion of *Orly *also has no coding potential. Further electronic searching of the various *Orly *transcriptional variants revealed no significant open reading frames other than a partial degenerate retroviral *pol *sequence (see below).

### Orly retains potential promoter sequence from both *Ssty1 *and *Sly*

The regions of high sequence identity between *Orly *and its various progenitor loci extend a further 5 kb into the upstream region of *Ssty*, and 3 kb into the upstream region of *Sly*, indicating that the rearranged locus has retained the proximal upstream promoter regions of both genes, in antisense orientation relative to each other (Additional File [Supplementary-material S1]). *Orly_v1 *and *Orly_v2 *are known to be transcribed from the relict *Ssty1 *promoter [16], which is thus shown to be functional.

Turning to the relict *Sly *promoter region, we conducted a search for transcription factor binding sites using TFSEARCH [17]. This showed that of the 42 predicted transcription factor binding located between -600 and +10 of the reference *Sly *sequence, 35 were present at the corresponding site in *Orly*, indicating retention of potentially functional promoter elements (Additional File 2). Overall sequence identity between *Sly *and *Orly *across this region is 95.7%. Significantly, the conserved elements include a GCCAAT box at position -161 of the reference *Sly *locus. This motif is a strong transcriptional signal, and is known to be present in other spermatid specific TATA-less promoters such as the *Pgk-2 *promoter [18].

### Both of the promoters at opposite ends of *Orly *are functional

This electronic promoter analysis suggested to us that the relict *Sly *promoter at the 3' end of the *Orly *locus may have retained functionality, and be able to generate opposite-strand transcripts. We designate such opposite-strand transcripts as *Orlyos*. No *Orlyos *transcripts were present in the nr or dbEST databases. We used strand-specific RT-PCR to determine whether any of the bands shown in Figure 2 corresponded to *Orlyos *transcripts.

**Figure 4:**
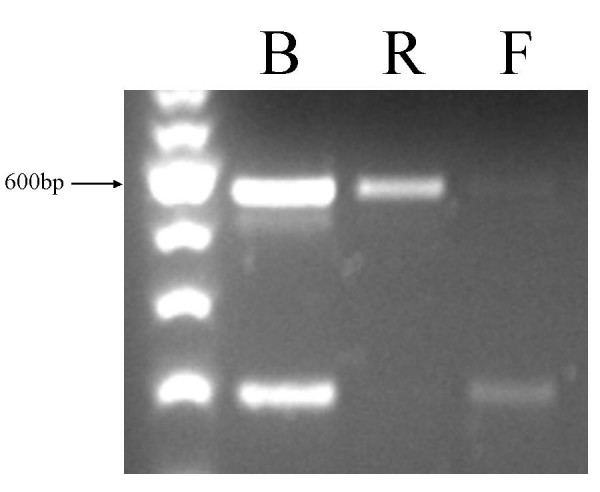
RT-PCR on adult testis using primer pair N1.f/N2.r2. B: Both primers present during RT step, both products are detected. R: RT reaction was primed with N1.f and detects a "reverse" transcript. F: RT reaction was primed with N2.r2 and detects a "forward" transcript.

Importantly, of the three bands obtained using the N1.f/N2.r2 primer pair, the lower band corresponds to a forward orientation (*Orly*) transcript, while the upper band corresponds to a reverse orientation transcript (Figure 4). Sequencing confirmed that this RT-PCR product shows 99% identity to the reference *Orly *genomic sequence over 622 nt, and only 91% identity to *Sly *over 464 nt. This demonstrates that this transcript arises from an *Orly *locus rather than from *Sly*, and constitutes a true *Orlyos *transcript. The middle band seen in this reaction is not always detected by RT-PCR, and we were unable to determine the direction of transcription of this band.

Of the other splice variants shown in Figure 2, all were confirmed by strand-specific RT-PCR to be "forward" (*Orly*) transcripts (data not shown). This is unsurprising as the primers were designed against the forward transcript *Orly_v2*. *Orlyos *transcripts must necessarily have different exon boundaries, which presumably do not include the majority of the primer locations included in our screen.

### The terminal exons of *Orly *derive from a retrovirus and are in antisense to *Sly *and Orlyos

Both of the known *Orly *transcripts terminate with two novel exons with antisense homology to intron 2 of *Sly *(see Figure 1A, exons N1 and N2). As discussed above, this section of *Orly *is also transcribed in the opposite orientation, generating *Orlyos *transcripts. Thus there is the potential for *Orly *transcripts to form dsRNA either by pairing with *Orlyos *transcripts or with nascent *Sly *transcripts.

Exons N1 and N2 derive from a partial degenerate retrovirus belonging to the MuRVY lineage of mouse Y chromosome specific repeats [19], which is embedded in this intron of *Sly *(see below). *Orly-F *transcripts terminate at the transcription stop site of this MuRVY-related element. We therefore deduce that although the MuRVY element is degenerate and does not encode a functional retrovirus, its transcriptional termination site has remained functional and become co-opted to form the transcriptional termination site for *Orly *forward transcripts. None of the known *Orly *transcripts contain any large open reading frames, however, *Orly_v1 *contains a short ORF running from bases 117–284. This ORF has 63% identity and 75% similarity over 49aa to a partial *pol *gene (data not shown), further demonstrating the retroviral origin of the terminal exons of *Orly*.

### Tissue- and developmental stage-specific expression of *Orly *isoforms

*Orly *transcripts are under tight transcriptional control. All variant forms (both forward and reverse) are only observed after day 19 of postnatal life, and thus are deduced to be spermatid-specific. This is to be expected as both *Ssty1 *and *Sly *promoters are spermatid specific [9,13,16]. The age of first appearance for each band varied from day 19 to 23, indicating differential regulation of *Orly *isoforms in successive spermatid stages. This variation was observed both between different primer pairs (e.g. the majority of S1.f1/A4.r bands appear at 23 dpp, while the majority of S1.f1/A3.r bands appear at 21 dpp), and between different bands detected by the same primer pair (e.g. the upper, lower and middle bands in the N1.f/N2.r2 reaction appear at 19, 21 and 23 days respectively). This differential regulation may be due to spermatid stage dependent splicing of transcripts, or may represent varying subsets of transcripts arising from different copies of *Orly *with subtly different promoter activities. It is unfortunately not possible to use *in situ *or Northern blot data to confirm the detailed cellular expression patterns of these transcripts, since there is no portion of any of them which is not also part of a different Y-linked gene or retrovirus with a confounding expression pattern.

### Genomic comparisons of *Orly*, its progenitor loci, and their X homologues

We carried out a detailed comparison of the genomic loci encoding *Orly*, the other MSYq genes and their X counterparts, in order to better delineate the sequence of events during MSYq evolution.

### Genomic comparison of *Ssty1 *and *Ssty2*

**Figure 5:**
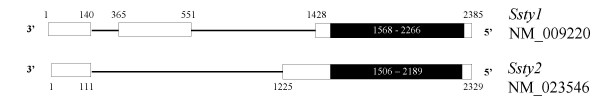
Genomic arrangement of *Ssty1/2*. Numbers indicate nucleotide position within the genomic locus. White boxes indicate exons, with the coding region shown in black.

The structure of the reference genomic loci encoding *Ssty1/2 *is shown in Figure 5 (see Additional File 3 for ClustalW alignment). Sequence identity is 82.7% across the locus as a whole. The coding region is conserved (barring the final 5 amino acids of *Ssty1*), and lies entirely within exon 3. Sequence identity within the coding region is 86.4%. The splice site at the end of the first exon is conserved. *Ssty2 *does not incorporate sequence corresponding to exon 2 of *Ssty1*. The splice site at the start of the terminal exon is located differently in *Ssty1 *and *Ssty2*, the latter thus having a longer terminal exon which in part matches intronic sequence from *Ssty1*. The 5'UTRs of the two gene transcripts are thus very different despite the high sequence identity between the loci. An X-linked member of this gene family (*Sstx*) is known, however, the similarity at the nucleotide level is low except for a small segment at the start of the coding region (76% over 135 bp) [Paul Burgoyne, personal communication].

### Genomic comparison of *Xmr *and *Xlr*

**Figure 6:**
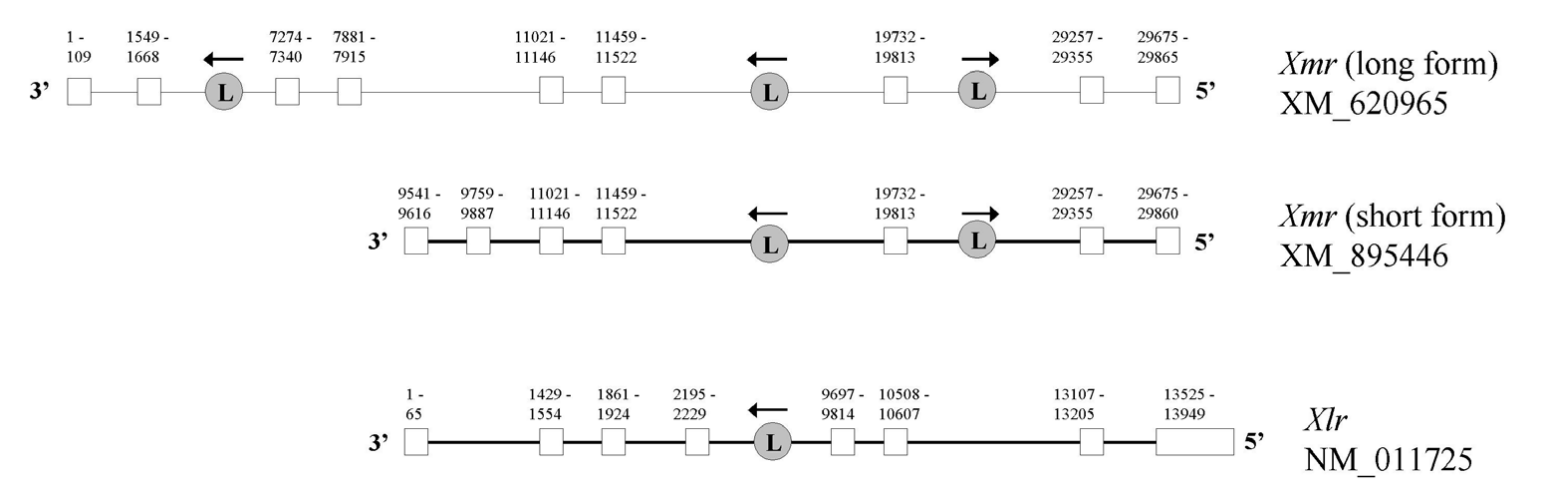
Genomic arrangement of *Xmr *and *Xlr*.  indicates the position and orientation of LINE elements. *Xmr *contains a LINE insertion in intron 7 that is not present in *Xlr *and presumably postdates *Xmr/Xlr *divergence. There are two transcriptional variants of *Xmr*, neither of which splice in an exon corresponding to *Xlr *exon 5. This exon encodes the nuclear localisation signal, thus *Xmr *has lost the ancestral nuclear pattern of expression. The longer *Xmr *isoforms has 4 unique 5' exons.

The structure of the reference genomic loci for *Xmr *and *Xlr *is shown in Figure 6. *Xmr *has two transcriptional variants arising from alternative transcription start sites, as indicated in this figure. The structure of *Xlr *is similar to the shorter transcriptional variant of *Xmr*, however, there is considerable sequence divergence in exon 4 of *Xlr*. In particular, the 3' portion of *Xlr *exon 5 does not match any *Xmr *sequence. The longer transcript derived from the *Xmr *locus contains 4 additional 5' exons which do not show sequence similarity to the upstream region of the *Xlr *locus. These exons thus represent either novel sequence acquired by *Xmr *following *Xmr/Xlr *divergence, or deletion of the 5' portion of the *Xlr *locus.

There are two partial degenerate LINE elements within the *Xmr *locus, the first lying in the second intron of the longer isoform, and the second lying in the sixth intron (and thus also present in the fourth intron of *Xlr*). In addition to these degenerate LINEs, *Xmr *also contains a full-length LINE element from the L1MD-A2 lineage, which includes upstream monomer repeats and thus is potentially transcriptionally active [20]. The element lies in intron 7 of *Xmr *but is not found in the corresponding location (intron 6) of *Xlr*, indicating that the LINE insertion occurred subsequent to *Xmr/Xlr *divergence.

### Sly arose as a chimeric gene via fusion of *Xmr *and *Xlr*

**Figure 7:**
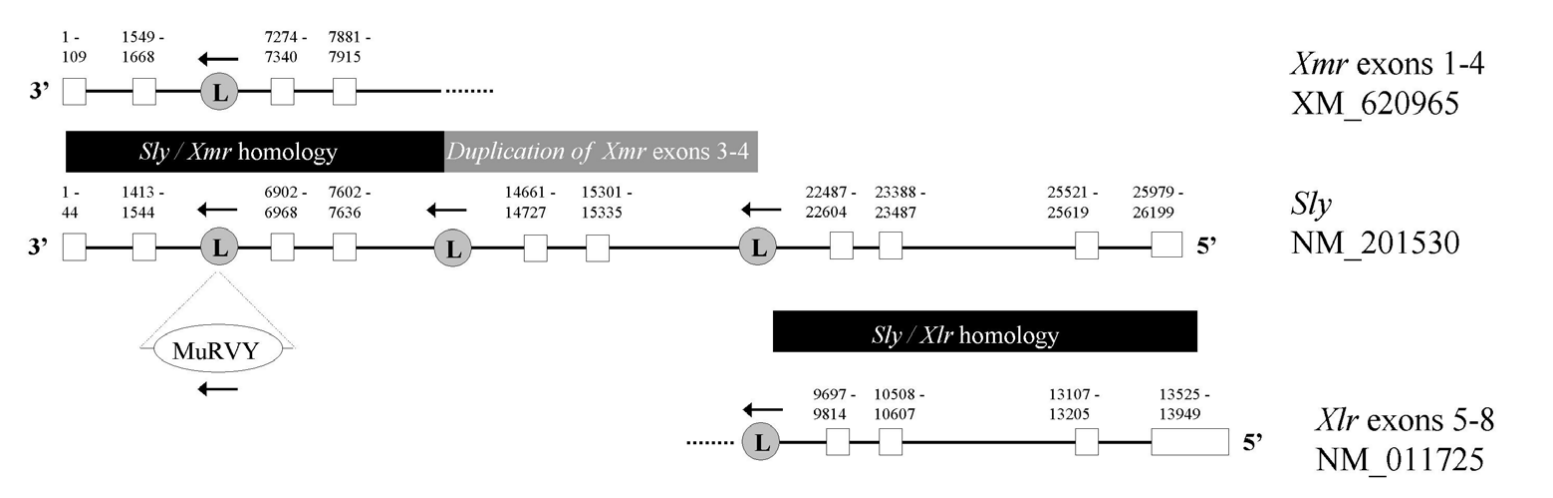
Genomic arrangement of *Xmr*, *Xlr *and *Sly*. *Sly *exons 1–4 are homologous to *Xmr *exons 1–4. *Sly *exons 5–6 represent an internal duplication of exons 3–4. *Sly *exons 7–10 are homologous to *Xlr *exons 5–8. There are LINE elements at the borders of each section, suggesting that *Sly *was formed by recombination between LINE repeats.  indicates the location and orientation of these LINE elements.

*Sly*, the Yq-linked member of the family, is a chimeric gene formed by fusion of the 5' portion of *Xmr *to the 3' portion of *Xlr*. Figure 7 shows the structure of *Sly *together with the homologous regions of *Xmr *and *Xlr*.

The origin of the 5' end of *Sly *is demonstrated by exons 1–4, which match the 4 additional exons uniquely present in the longer *Xmr *isoform. The origin of the 3' end of *Sly *is demonstrated by exons 7–10, which match the final exons of *Xlr *including the *Xlr*-specific portion of exon 5. *Sly *lacks the L1MD-A2 element present in *Xmr *intron 7, further confirming the chimeric nature of this gene as an *Xmr/Xlr *hybrid. Exons 5–6 of *Sly *arose via duplication of exons 3–4 and show 88/102 nucleotide identity to these exons. There are degenerate LINE elements at the borders of this duplication event, and also at the border between the *Xmr*-derived and *Xlr*-derived segments of *Sly*, thus it is likely that recombination between LINE elements was responsible for the creation of *Sly*.

The LINE element present in intron 2 of *Sly *is interrupted by a stretch of DNA with distant sequence similarity to the mouse MSYq-specific retrovirus, MuRVY. This LINE element is uninterrupted in the progenitor *Xmr*, thus we conclude that the MuRVY insertion occurred subsequent to the creation of *Sly*. The MuRVY-related sequence is inserted in antisense orientation relative to *Sly *itself. The extent of the MuRVY-related stretch of DNA varies between *Sly *copies (see phylogenetic tree analysis below), but in all cases the terminal portion (including MuRVY transcription termination site) is retained. RepeatMasker analysis [21] of the insert shows 13.1% divergence, 13.1% deletion and 3.2% insertion relative to the consensus MuRVY LTR sequence, and 32.5% divergence for non-LTR portions of the insert. As discussed above, the MuRVY-related sequence in *Sly *intron 2 forms the source for the terminal *Orly *exons.

Recent work has shown that both *Xmr *and *Sly *are cytoplasmic proteins, in contrast to *Xlr*, which is nuclear[22]. The KRKR nuclear localisation signal in *Xlr*, which is conserved from the autosomal progenitor gene SCP3, is located in exon 5. This signal is mutated to KRKW in the corresponding portion of *Sly*, while *Xmr *does not include this exon at all. Thus while *Xlr *retains the ancestral nuclear pattern of protein localisation, both *Xmr *and *Sly *have become cytoplasmic via different mechanisms.

### Genomic comparison of *Asty/Astx*

As reported previously [16], *Asty *and *Astx *have an identical genomic organisation, and share ~95% sequence identity across introns and exons.

### The genomic context of *Orly*

We used BLAST comparison to search for all copies of each Yq-linked gene (*Ssty1/2*, *Asty*, *Sly*, *Orly*) in the currently-released draft sequence contigs [Mouse Chromosome Y Mapping Project (Jessica E. Alfoldi, Helen Skaletsky, Steve Rozen, and David C. Page at the Whitehead Institute for Biomedical Research, Cambridge MA, and the Washington University Genome Sequencing Center, St. Louis MO)].

We then used this information to generate a "fingerprint" for each available Yq contig, noting the order and orientation of the various copies of each gene present in each contig (see Additional File 4). Interestingly, we found that *Orly *always has the same genomic context, being flanked downstream by *Ssty1 *and upstream by *Ssty2*, with both loci in the same orientation as *Orly*. The neighbouring copies of *Ssty1 *all contain a SINE insertion at position 393, and form a distinct sub-group within the phylogenetic tree (see below: bootstrap support value for this clade is 1000/1000 replicates).

Using the fingerprints as a guide, we were able to assemble a "super-contig" containing 3 copies of *Ssty1*, 3 copies of *Ssty2*, two copies of *Asty*, two copies of *Sly *and one copy of *Orly*. In all, 13 of the 33 Yq contigs are congruent with this super-contig ordering, and a further 4 contigs appear to be slight variants upon it. This "super-contig" indicates the presence on mouse Yq of a highly amplified repeat unit of greater than 500 kb in length, which presumably corresponds to the Huge Repeat Array reported at conferences by Alfoldi *et al *[23]. Sequence identity between the various contigs contributing to this "super-contig" is very high (> 98% excluding indels), indicating substantial homogeneity between copies of the Huge Repeat.

**Figure 8:**
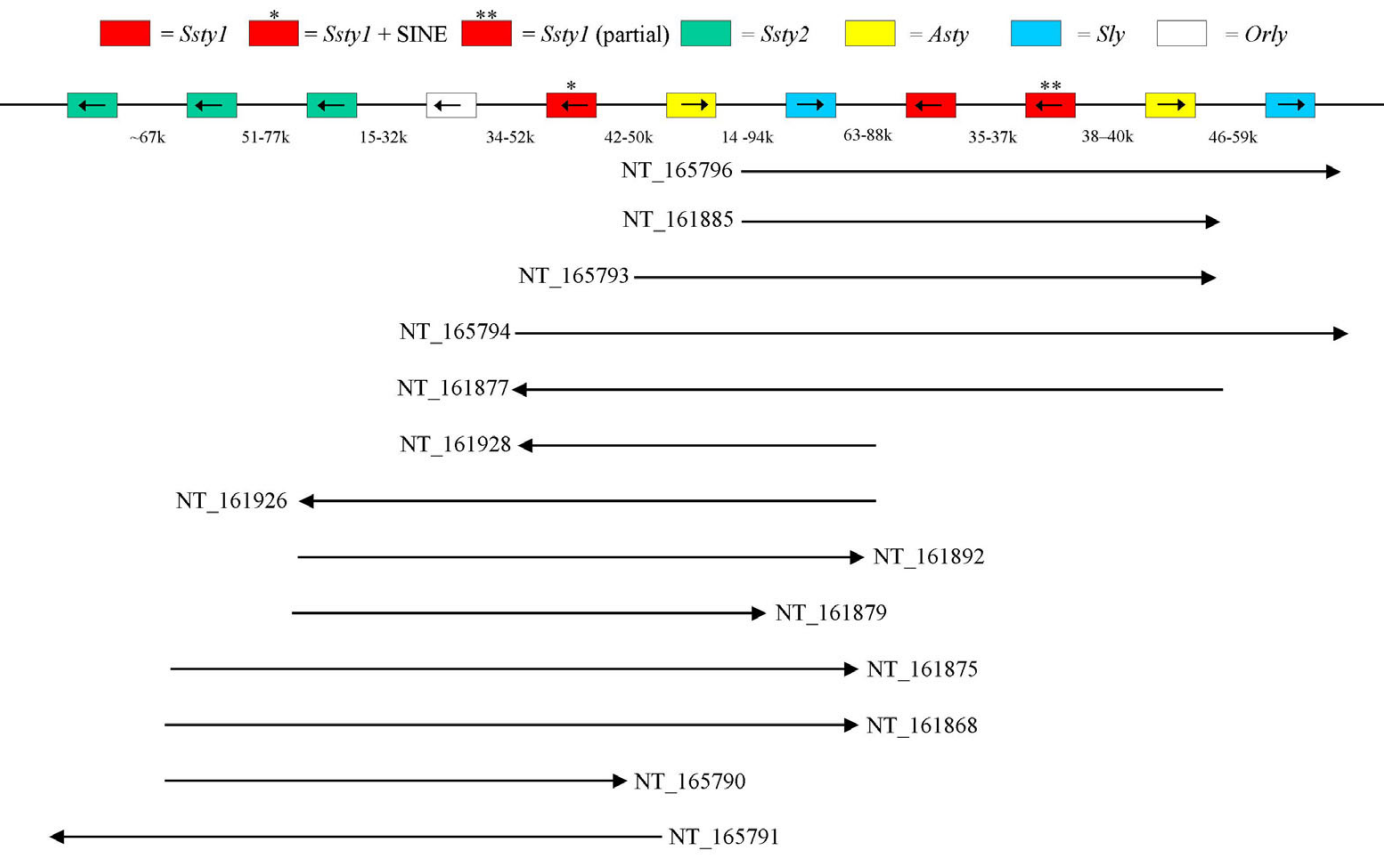
Schematic diagram showing the layout of the Huge Repeat Array repeat unit on mouse Yq. Order and orientation of Yq gene copies is shown, with individual contigs represented as line segments below. The size range of the interval between each gene locus is shown. The repeat unit is at least 500 kb in length, but our current analysis is unable to define the complete extent of the repeat. The *Ssty1 *copies within the repeat can be distinguished from each other based on the presence or absence of a SINE insertion, and by a deletion removing exons 1–2 of one copy of *Ssty1 *(see also **Figure 10**).

**Figure 9:**
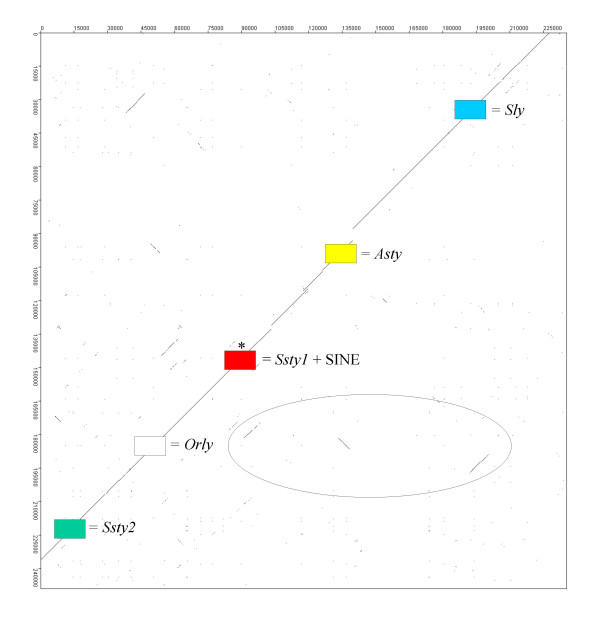
Dot plot comparing [GenBank:NT_161892] to [GenBank:NT_161926]. This demonstrates the very high degree of sequence identity between copies of the Huge Repeat. Locations of gene loci within the contigs are indicated. The homology between *Orly *and its component loci is clearly visible as three short internal repeat sections within the contigs (ringed).

**Figure 10:**
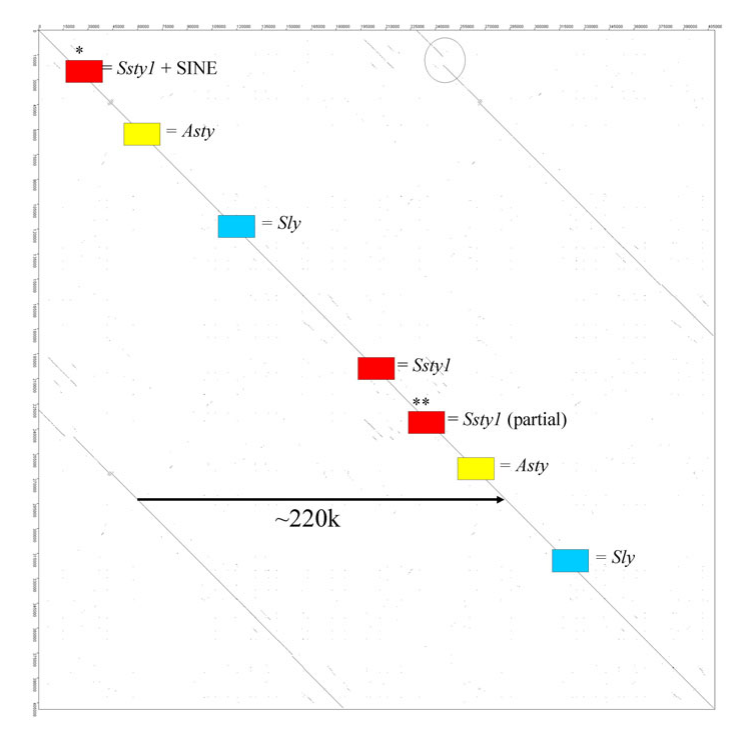
Dot plot comparing [GenBank:NT_165794] to itself, in order to demonstrate the nested repeat structure of the Huge Repeat Array. Although the repeat unit of the Huge Repeat Array spans at least 500 k (see **Figure 8**), it also contains an internal repeat with a unit length of ~220k, containing copies of *Ssty1*, *Asty *and *Sly*. Visible (ringed) is a small deletion in one of the repeat units, which has deleted the first two exons of the *Ssty1 *copy located in the region.

Figure 8 shows the layout of the Huge Repeat unit, and the contigs that match this ordering. Figure 9 is an example Dotter alignment of two Huge Repeat contigs, [GenBank:NT_161892] and [GenBank:NT_161926], demonstrating that high sequence homology extends across the entire contig, genic and intergenic regions included. In all, these two contigs share 98.26% nucleotide identity over 235813 nt. The Huge Repeat is itself internally repetitive – it can be seen in Figure 8 that there is a repeating segment containing *Ssty1*, *Asty *and *Sly *in order. Figure 10 is a Dotter plot comparing [GenBank:NT_165794] to itself, demonstrating this internal repeat. The repeat region in this contig shows 98.12% identity over 179584 nt.

**Figure 11:**
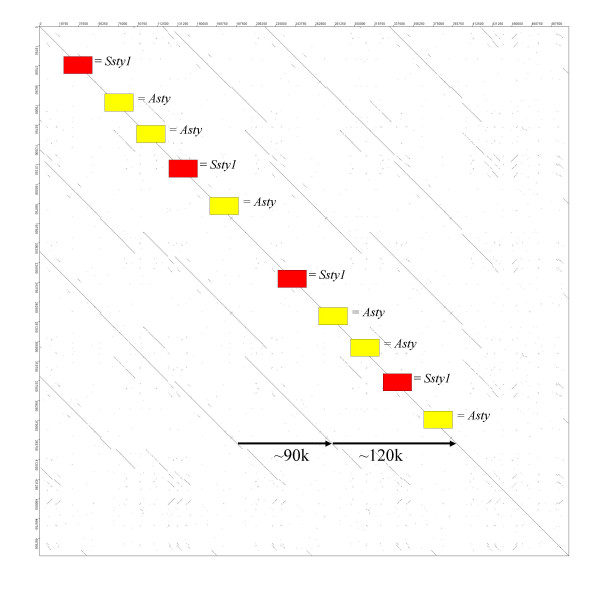
Dot plot comparing [GenBank:NT_161904] to itself. This contig is not part of the Huge Repeat, but forms part of an *Ssty1*/*Asty *enriched subset of Yq contigs. This class of Yq sequence also appears to have been amplified in recent mouse evolutionary history, concurrently with Huge Repeat Array expansion. This contig contains nested direct repeats of ~90 k and ~120 k, giving a total repeat length of ~210 k.

However, many contigs did not fit the Huge Repeat consensus ordering. Two further classes of Yq contig were identified (see Table 2): *Ssty*/*Asty*-enriched contigs (n = 7) and *Sly */*Asty*-enriched contigs (n = 5), while 4 contigs remained unclassifiable. These non Huge Repeat contigs were also highly internally repetitive. Figure 11 is an example Dotter plot comparing [GenBank:NT_161904] to itself. The structure is of a tandem repeat unit of ~120 kb embedded inside a larger tandem repeat of ~210 kb. In all, the repeated segment in NT_161904 shows 97.78% identity over 204851 nt.

**Table 2:** Classes of contig identified on mouse Yq

**Huge Repeat**	[GenBank:NT_161868, GenBank:NT_161875, GenBank:NT_161877, GenBank:NT_161879, GenBank:NT_161885, GenBank:NT_161892, GenBank:NT_161926, GenBank:NT_161928, GenBank:NT_165790, GenBank:NT_165791, GenBank:NT_165793, GenBank:NT_165794, GenBank:NT_165796]
**Similar to Huge Repeat**	[GenBank:NT_161923, GenBank:NT_161924, GenBank:NT_161937, GenBank:NT_165792]
***Ssty/Asty*-enriched**	[GenBank:NT_161897, GenBank:NT_161898, GenBank:NT_161904, GenBank:NT_161919, GenBank:NT_165795, GenBank:NT_165797, GenBank:NT_165798]
***Sly/Asty*-enriched**	[GenBank:NT_161866, GenBank:NT_161872, GenBank:NT_161913, GenBank:NT_161916, GenBank:NT_161925]
**Unclassifiable**	[GenBank:NT_161895, GenBank:NT_161902, GenBank:NT_161906, GenBank:NT_161911]

Contig classes are defined by contig content in terms of the known Yq-linked genes. The Huge Repeat consensus ordering of gene loci is shown in Figure 8.

### Dynamics of Yq gene family expansion

A key question is whether these four gene families (*Ssty1/2, Asty, Sly *and *Orly*) were amplified separately on Yq during mouse evolution, or whether there was a single period of amplification increasing the copy number of all genes simultaneously.

**Figure 12:**
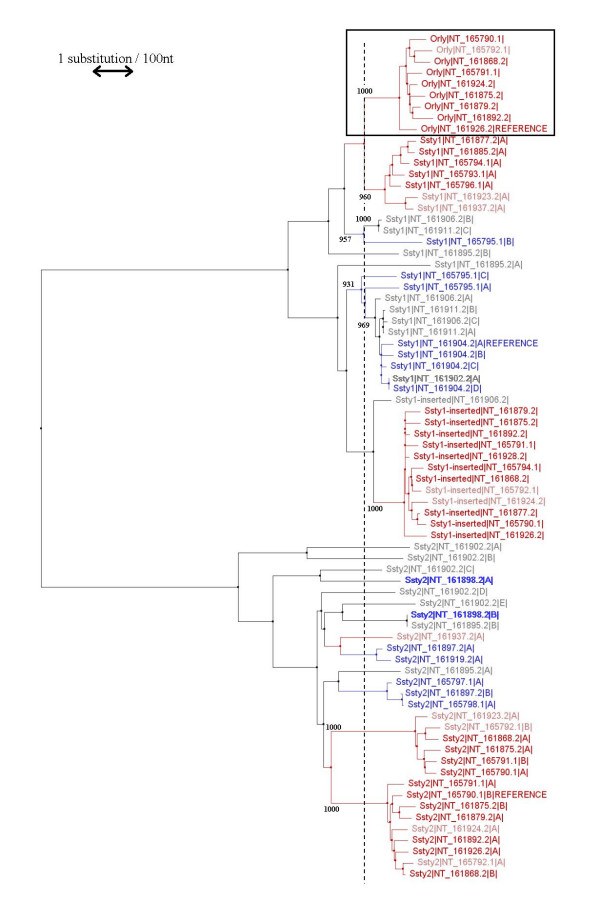
Phylogenetic tree of *Ssty1*, *Ssty2 *and *Orly*. Colours indicate which class of Yq contig each gene copy is located in (see **Table 2**). Red = Huge Repeat contig. Pink = contigs with fingerprints similar but not identical to the Huge Repeat. Green = *Sly/Asty*-enriched contigs. Blue = *Ssty/Asty*-enriched contigs. Grey = unclassifiable contigs. The scale bar indicates 1 substitution per hundred base pairs of sequence. Numbers indicate bootstrap support values (out of 1000 replicates) for key clades discussed in the text. The *Orly *clade is boxed. The dashed line indicates the point at which *Orly *was created. The intersection of this line with the branches of the tree shows how many lineages of each gene were present at the time of *Orly *divergence.

**Figure 13:**
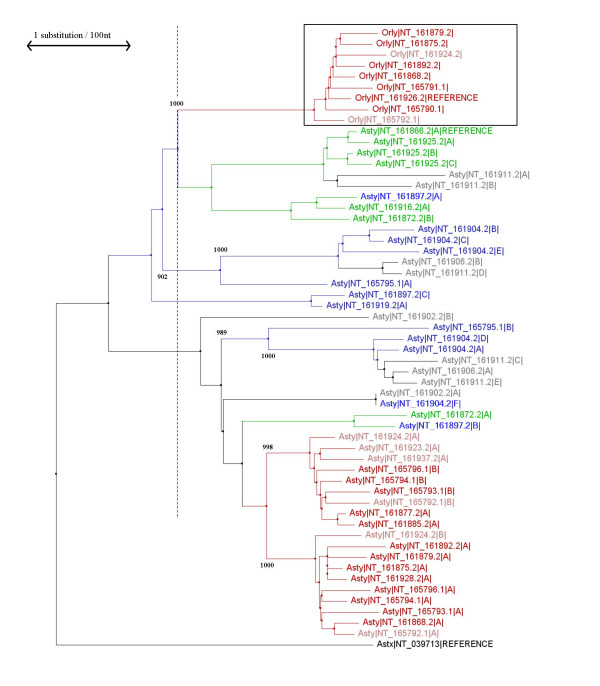
Phylogenetic tree of *Asty *and *Orly*. *Astx *is also included, and was used as the outgroup to root the tree. Colours, scale bar etc. are as in **Figure 12**.

**Figure 14:**
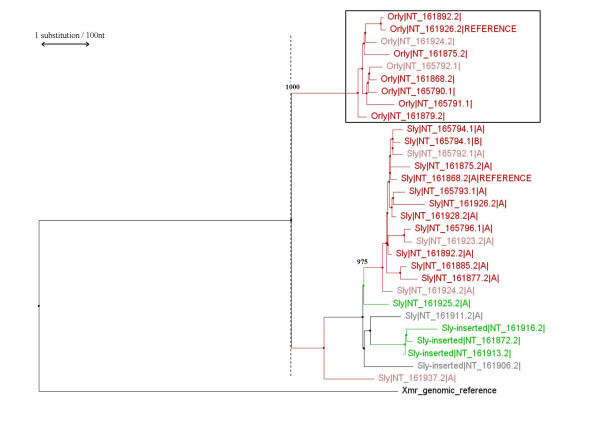
Phylogenetic tree of *Sly *and *Orly*. *Xmr *is also included, and was used as the outgroup to root the tree. Colours, scale bar etc. are as in **Figure 12**.

We constructed a set of phylogenetic trees using the neighbour-joining method of Saitou & Nei [24], comparing *Ssty1-, Asty- *and *Sly-*derived regions of *Orly *to the corresponding regions of the progenitor loci (Figures 12, 13, 14). For the *Asty- *and *Sly*-related trees, we were able to use the X-linked homologue as an outgroup to root the tree, however, for the *Ssty-*related tree this is not possible owing to much greater divergence of the X homologue which precludes accurate alignment. For this tree, the *Ssty2 *clade was used as the outgroup to root the tree. Bootstrap analysis of 1000 replicates was used to establish the robustness of all three trees.

From this phylogenetic analysis we observe:

• In all three cases, *Orly *sequences form a discrete clade (bootstrap support value of 1000/1000 replicates for all three trees).

• Gene copies lying within the Huge Repeat contigs also form distinct clades in all three trees (bootstrap support of 960/100 to 1000/1000 in all cases). Note that each copy of the Huge Repeat unit contains several copies of *Ssty1*, *Ssty2 *and *Asty*. These three genes thus give rise to several Huge Repeat-associated clades in each tree. Each of these clades contains the gene copies from matching locations within the Huge Repeat unit.

• A final set of contigs forms a distinct clade in both the *Ssty *and *Asty*-related trees. This clade contains a group of *Ssty1 */*Asty*-enriched contigs, [GenBank:NT_161904], GenBank:NT_161906, GenBank:NT_161911] (bootstrap values 969/1000 to 1000/1000 in the two trees). At slightly lower confidence levels, this clade also includes [GenBank:NT_165795] (bootstrap values 902/1000 to 989/1000 in the two trees).

• At the time of *Orly *divergence, the *Ssty *family was already moderately amplified on the Y, with ~8 *Ssty1 *lineages and ~13 *Ssty2 *lineages present. By contrast, at the time of *Orly *divergence, there were only ~4 *Asty *lineages and 1 *Sly *lineage present on the Y

• In all three cases, there was a massive amplification of gene copy number subsequent to *Orly *divergence. This amplification occurred predominantly in branches of the phylogenetic tree corresponding to Huge Repeat contigs, however, there was also amplification of a *Ssty1 */*Asty*-enriched clade subsequent to divergence of the *Orly *clade.

From these trees, we also observed that all genes within each family showed very similar degrees of divergence from the root of the tree in all cases. This is to be expected as all three trees were based on noncoding sequence. The sequence used to build the trees is thus likely to be evolving at nearly neutral rates. Given nearly neutral rates of evolution, the degree of sequence divergence forms a "molecular clock" indicating the timing of the various events on mouse Yq. We therefore also generated trees using the UPGMA algorithm, which explicitly assumes a molecular clock (Additional Files 5, 6, 7).

In this analysis, the percentage divergence of *Orly *from its progenitor loci (representing the date of generation of *Orly*) is 1.24% for *Orly*/*Ssty1*, 1.79% for *Orly*/*Asty *and 1.87% for *Orly*/*Sly*. The percentage divergence between the *Orly *branches of the tree (representing the date of amplification of the Huge Repeat Array) is 0.47% for the *Ssty1-*derived region, 0.41% for the *Asty-*derived region and 0.43% for the *Sly-*derived region. While the absolute rate of the clock cannot be determined from these data, the numbers obtained from the three trees are in good agreement with each other, strengthening our inferences of the timing of events on Yq.

### Conclusions of the phylogenetic study

Taken together, these results of the phylogenetic tree analysis and locus fingerprinting of Yq contigs indicate that events on Yq occurred in the following sequence.

1) *Sstx*/*Ssty *divergence (too long ago to be addressed by nucleotide sequence analysis)

2)*Ssty1*/*Ssty2 *divergence

3) Generation of *Sly *by chimerism between *Xmr *and *Xlr*

4) Moderate amplification of *Ssty1*, *Ssty2 *and *Asty*

5) Generation of *Orly *by chimerism between *Ssty1*, *Asty *and *Sly*

6) Massive amplification of two familes of large-scale repeat on Yq. The first repeat family contains representatives of all Yq genes including *Orly *and constitutes the Huge Repeat Array, while the second specifically contains *Ssty1 *and *Asty*.

At present unresolved is the question of when the MuRVY retrovirus arrived on Yq. The presence of MuRVY-related sequence within intron 2 of every copy of *Sly *indicates that *Sly *acquired its MuRVY-derived insert in intron 2 some time between stages (3) and (6), however, the origin of MuRVY itself cannot be placed in the above sequence from available evidence.

## Discussion

We report here on the genomic locus *Orly *and the wide variety of alternatively spliced transcripts arising from it. *Orly *has a complex and unusual genomic structure, being derived from partial copies of three other Yq-linked genes. Intriguingly, we also found *Sly *to be derived by combination of existing genes, in this case a fusion of the 5' region of *Xmr *with the 3' region of *Xlr*, together with an internal duplication of exons 3–4 of the *Xmr*-derived segment. This may indicate that chimerism and "exon shuffling" are a general feature of novel Y chromosome gene creation. Significantly, the two outermost partial gene loci contributing to *Orly *are in antisense orientation relative to each other, and retain their upstream promoter regions. We detected *Orlyos *transcripts in addition to *Orly *transcripts, and thus deduce that both promoters have retained their activity. In particular, exons N1, N2 and the intervening intron are transcribed in both directions. This region derives from a MuRVY retroviral insertion into intron 2 of *Sly*.

There is an intriguing parallel to be drawn with the *Stellate *system in *Drosophila melanogaster*, where there is a sense/antisense regulatory loop between X-encoded *Stellate *and Y-encoded *Su(Ste) *repeat genes [25]. In the case of *Stellate*, the Y gene arose from the X gene by insertion of a transposon (with active promoter) in reverse orientation [26,27]. Antisense *Su(Ste) *transcripts primed from the transposon promoter act to regulate both sense *Su(Ste) *and *Stellate *transcript levels via an RNAi mechanism [25,28]. Similarly, *Orly *and *Orlyos *transcripts could potentially regulate each other and also *Sly*. A key avenue of future work is to determine the full length sequence of *Orlyos*, in particular whether it contains any *Ssty1- *or *Asty-*derived regions which may in turn regulate these genes.

The comparison to *Stellate *is especially interesting given the sex ratio skewing in male mice bearing partial Yq deletions. Partial deletions of the repressor *Su(Ste) *on *Drosophila *Y chromosome lead to sex ratio skewing or infertility dependent upon the X chromosomal *Stellate *haplotype present [29]. *Stellate *was hypothesised to be a meiotic drive gene [30,31], although this is now disputed [32]. In male mice, partial deletions of Yq lead to mild teratozoospermia and sex ratio skewing [9,11,14], with reduced effectiveness of Y-bearing sperm [15]. Larger deletions lead to severe teratozoospermia and infertility [8,13]. The mice with partial deletions show normal fertility and fecundity (in terms of number of successful matings and number of offspring per litter), thus the only effect of the decrease in Yq gene copy number appears to be the sex ratio skew.

It should be understood that the sex ratio skew in mouse with Yq deletions does not constitute meiotic drive in the classical sense, since equal numbers of X- and Y-bearing gametes are generated at meiosis [15]. Nevertheless, the presence of Yq-encoded genes affecting sex ratio indicates the potential for a conflict between these Yq-encoded genes and other interacting X- or autosomally-encoded factors. Given that Yq deletion also leads to a spermatid-specific derepression of X transcripts [33], with increasing X gene expression correlated with the extent of the deletion, we have suggested that there may indeed be an ongoing genomic conflict between the mouse X and Y chromosomes, with X-linked sex ratio distorter genes acting to favour generation of female offspring, and Yq-linked repressor genes acting to restore a normal 50:50 sex ratio. Such an intragenomic conflict is expected to lead to massive amplification of gene number on both chromosomes due to an "arms race" between the conflicting genes [34]. Intriguingly, the hybrid sterility seen in *Mus musculus musculus*/*Mus musculus molossinus *consomic strains is X-dependent [35].

Whether genomic conflict is involved or not, the fact that Yq-encoded genes are necessary for normal levels of Y chromosome transmission necessarily leads to a strong and direct evolutionary pressure to maintain the function of these genes. This may be one of the factors behind the recent and highly unusual gene amplification seen on mouse Yq. *Orly*, being composed of portions of all the other known MSYq-linked genes, must also necessarily be the most recent known addition to MSYq gene content.

## Conclusion

*Orly *is a novel chimeric locus on mouse chromosome Yq which is bidirectionally transcribed, giving rise to *Orly *and *Orlyos *transcripts. These transcripts may potentially form dsRNA in partnership with each other, or with the progenitor loci *Ssty1*,*Asty *and *Sly*. A phylogenetic tree analysis of Yq genes indicates that *Orly *arose shortly prior to a massive expansion in copy number of all the Yq-linked genes. Also, potentially significantly, copies of *Orly *are only found in the context of the Huge Repeat Array that distinguishes MSYq – a particular segment of around 500 kb that appears to have been amplified *en bloc*. Taking the above evidence together, we propose that the emergence of *Orly *may have been one of the triggers that led to massive amplification of Yq sequence. Further analysis of the genomic complement of MSYq, and the copy number of the corresponding X genes, in a range of different mouse subspecies should help date these events more precisely, and establish whether X-Y genomic competition is a contributing factor to the gene amplifications.

## Methods

### Sequence comparison and detection of copies of Yq-linked genes

Nucleotide sequence alignment was performed using BLAST and ClustalW. Copies of Yq-linked genes were located within the currently-released draft sequence contigs [Mouse Chromosome Y Mapping Project (Jessica E. Alfoldi, Helen Skaletsky, Steve Rozen, and David C. Page at the Whitehead Institute for Biomedical Research, Cambridge MA, and the Washington University Genome Sequencing Center, St. Louis MO)] by pairwise alignment of reference gene sequences to each contig. All full-length hits were recorded, thus this study does not distinguish between genes and pseudogenes in each family. For *Ssty1, Ssty2*, *Asty *and *Orly*, a window size of 40 nt was used, while for *Sly *a window size of 100 nt was used. Additional File 4 is a complete record of all loci detected in the course of this study. The reference sequences used for this search were as follows.

*Ssty1*: ref|NT_161904.2|MmY_159612_36:c34361-31977

*Ssty2*: ref|NT_165790.1|MmY_163304_36:c172129-169801

*Sly*: ref|NT_161868.2|MmY_159576_36:275482-301680

*Asty*: ref|NT_161866.2|MmY_159574_36:c99035-96672

*Orly*: ref|NT_161926.2|MmY_159634_36:179263-194146

The reference sequences for *Ssty1*, *Ssty2 *and *Sly *are drawn from the Gene database of the NCBI [36]. The reference sequence for *Asty *was selected as the hit with the highest percentage identity to the known partial cDNA sequence [GenBank:DQ874391]. In the case of *Orly*, we define the locus as extending from the transcriptional start site (TSS) of the relict *Ssty1 *partial sequence to the TSS of the relict *Sly *partial sequence. The locus chosen as a reference is that encoding the known transcript *Orly_v1 *([GenBank:AK015935]). Note that both the reference genome sequence and the reference gene sequences are from the C57/Bl6 strain. Dot plots of selected contigs and gene loci were generated using JDotter [37], with grey scale values set to highlight the appropriate homologies.

### Phylogenetic tree analysis

All full-length copies of *Ssty1/2*, *Sly*, *Asty *and *Orly *identified by the contig search were used to build these trees. The reference sequences for *Xmr *and *Astx *were included in the appropriate trees in order to determine the timing of MSYq events relative to the split between X and Y homologues, however, the high degree of nucleotide sequence divergence between *Sstx *and *Ssty *precluded the inclusion of the X-linked gene for this tree.

For each gene family, a region excluding known protein-coding sequence was selected for alignment, thus nearly neutral rates of evolution can be assumed. Since *Asty *appears to be non-coding, the full length of all detected *Asty *sequences (~2.1 k) was used for the *Astx/Asty/Orly *tree, together with the homologous regions of *Astx *and *Orly*. For the *Ssty1/Ssty2/Orly *and for *Xmr/Sly/Orly *trees, the aligned region comprises the 3' UTR and all introns within the 3' UTR. This is ~1.5 kb for the *Ssty1/Ssty2/Orly *tree and 1.4 k for the *Xmr/Sly/Orly *tree.

Interestingly, the opening ATG codon was conserved in all detected copies of both families, including conservation of this codon at both ends of all copies of *Orly*. The significance of this observation is unclear. Alignment of the gene copies was performed using ClustalW via the EBI website [38], and ClustalX used to generate each tree using the Saitou/Nei NJ algorithm. 1000 bootstrap replicates were used to assess the robustness of each tree. Additional File 8 contains the three NJ trees and the ClustalW files used to generate each tree. JalView [39] was used to generate the figures included in this manuscript.

### RT-PCR

RNA samples were treated for DNA contamination using the RNAse free DNAse set (Qiagen). RT-PCR was performed using the One Step RT-PCR kit (Qiagen). Briefly, a reverse transcription step at 50°C for 30 minutes was followed by an activation step at 94°C for 15 minutes, and then 30 cycles of PCR at 94°C/T_m_/72°C for 10s/10s/30s. The annealing temperature T_m _varied from 53–55°C depending on primer combination. 23 *Orly *partial cDNA sequences detected in this work have been submitted to GenBank, accession numbers ES316436 to ES316458.

### Sequencing

Single-band RT-PCR products were purified using the Qiagen Qiaquick kit according to the manufacturers instructions. If multiple bands were present, these were gel purified using the Qiagen gel extraction kit. Purified RT-PCR products were sequenced from 5' and 3' ends using standard cycle sequencing methods.

## Competing interests

The author(s) declare that they have no competing interests.

## Supplementary Material

Additional file 1 Chimeric composition of the *Orly *locus. Dotter alignment showing the homologies between *Orly *and its constituent loci. Exon locations of the constituent loci are indicated, as are the novel exons contained in *Orly *transcripts. Click here for file

Additional file 2 Comparison of *Sly *upstream promoter region with homologous region of *Orly*. Annotated output from the TFSEARCH scan for potential transcription factor binding sites in the *Sly *upstream promoter region and the putative *Orlyos *promoter. Key elements such as the GCCAAT box are highlighted. Click here for file

Additional file 3 ClustalW alignment of *Ssty1 *and *Ssty2*. Annotated output from the ClustalW programme, aligning *Ssty1 *and *Ssty2*. Exons, coding region and the polyadenylation signal are highlighted for both genes. Click here for file

Additional File 4 Potential gene loci detected in Yq contigs. List of potential gene copies found within each genomic contig mapping to mouse chromosome Yq. Gene copies were located via BLAST comparison of reference gene loci to each contig. Start and end points (in bp) for each match are noted, as is the orientation of the potential gene copy, which may in each case be a coding gene or a pseudogene. Click here for file

Additional File 5 UPGMA Phylogenetic tree of *Ssty1*, *Ssty2 *and *Orly*. Phylogenetic tree of *Ssty1*, *Ssty2 *and *Orly *gene copies using the same alignment used for Figure 12. The UPGMA algorithm was used, rather than the neighbour-joining algorithm used for Figure 12. Click here for file

Additional File 6 UPGMA Phylogenetic tree of *Asty *and *Orly*. Phylogenetic tree of *Asty *and *Orly *gene copies using the same alignment used for Figure 13. The reference *Astx *sequence was used as the outgroup to root the tree. The UPGMA algorithm was used, rather than the neighbour-joining algorithm used for Figure 13. Click here for file

Additional File 7 UPGMA Phylogenetic tree of *Sly *and *Orly*. Phylogenetic tree of *Sly *and *Orly *gene copies using the same alignment used for Figure 14. The reference *Xmr *sequence was used as the outgroup to root the tree. The UPGMA algorithm was used, rather than the neighbour-joining algorithm used for Figure 14. Click here for file

Additional File 8 Clustal alignments used for tree analysis. ZIP archive file containing the ClustalW alignments used to generate the phylogenetic trees for this project (plain text format). Click here for file
