# Supplementary material for: Bidirectional transcription of a novel chimeric gene mapping to mouse chromosome Yq
Source: BMC Evol Biol. 2007 Sep 24;7:171. doi: 10.1186/1471-2148-7-171 (PMC2212661; doi:10.1186/1471-2148-7-171)
Supplement: Additional file 1 — Chimeric composition of the Orly locus. Dotter alignment showing the homologies between Orly and its constituent loci. Exon locations of the constituent loci are indicated, as are the novel exons contained in Orly transcripts. [file 1471-2148-7-171-S1.ppt]

## Slide 1
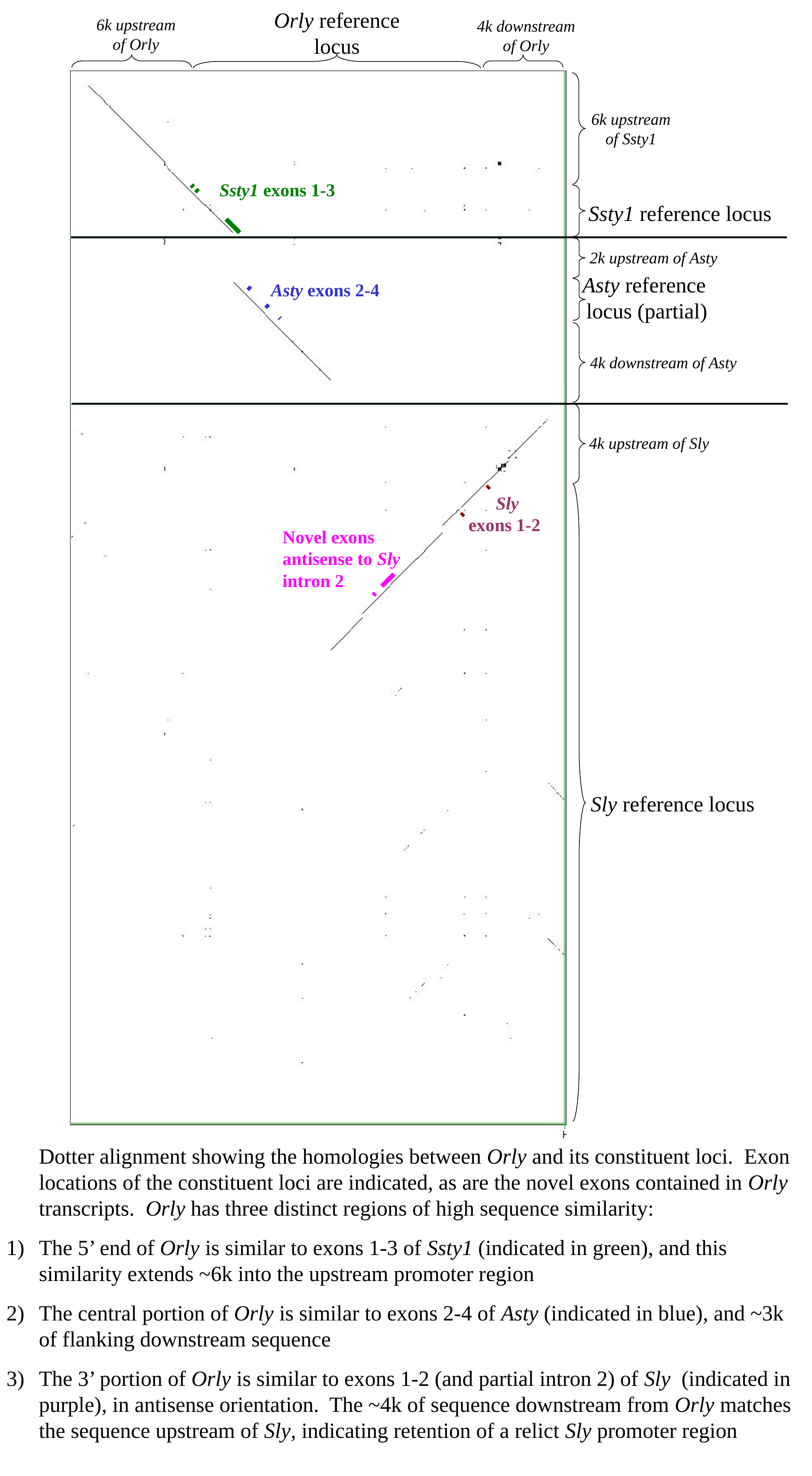

Orly reference locus
6k upstream
of Orly
4k downstream
of Orly
6k upstream
of Ssty1
Ssty1 exons 1-3
Ssty1 reference locus
2k upstream of Asty
Asty reference
locus (partial)
Asty exons 2-4
4k downstream of Asty
4k upstream of Sly
 Sly
exons 1-2
Novel exons antisense to Sly
intron 2
Sly reference locus
 Dotter alignment showing the homologies between Orly and its constituent loci. Exon locations of the constituent loci are indicated, as are the novel exons contained in Orly transcripts. Orly has three distinct regions of high sequence similarity:
The 5’ end of Orly is similar to exons 1-3 of Ssty1 (indicated in green), and this similarity extends ~6k into the upstream promoter region
The central portion of Orly is similar to exons 2-4 of Asty (indicated in blue), and ~3k of flanking downstream sequence
The 3’ portion of Orly is similar to exons 1-2 (and partial intron 2) of Sly (indicated in purple), in antisense orientation. The ~4k of sequence downstream from Orly matches the sequence upstream of Sly, indicating retention of a relict Sly promoter region
