# Supplementary material for: Bidirectional transcription of a novel chimeric gene mapping to mouse chromosome Yq
Source: BMC Evol Biol. 2007 Sep 24;7:171. doi: 10.1186/1471-2148-7-171 (PMC2212661; doi:10.1186/1471-2148-7-171)
Supplement: Additional file 2 — Comparison of Sly upstream promoter region with homologous region of Orly. Annotated output from the TFSEARCH scan for potential transcription factor binding sites in the Sly upstream promoter region and the putative Orlyos promoter. Key elements such as the GCCAAT box are highlighted. [file 1471-2148-7-171-S2.doc]

CLUSTAL W (1.83) multiple sequence alignment

Putative Sly Promoter GTGGCCAAGCACATAAGTGAGAGAGAGAGGGCCTGCACTGCCTAGCACACTCTTCTA-TC 59

Putative Orly Promoter GTGGCCAAGCACATAAGTGAGAGAGAGAGGGCCTGCACTGCATAGCACACTCTTCTAATC 60

***************************************** *************** **

<------ CdxA (shared)

<------------ HNF-3b (shared)

------> Nkx-2.5 (shared)

Putative Sly Promoter AAGCTGAGGACTAGGAAAGTAGAGAAAGGAGAGTACCAGGGGTTGTAGGAAATCTGCATG 119

Putative Orly Promoter AAGCTGAGGACTAGGAAAGTAGAGACAGGAGAGTACCAGGGGTTGAAGGAAATGTGCATG 120

************************* ******************* ******* ******

Putative Sly Promoter TTCCCTTCTGAACTCTTAGGGCAGCATAGACTCTTCTGGTGTGCGTGTGTACATGCAGAA 179

Putative Orly Promoter CTCCCTTCTGAACTCTTAGGGCAGCATAGACTCTTCTGGTGTGCGTGTGTACATGCAGAA 180

***********************************************************

<--------> HSF2 (shared)

<--------- NRF-2 (shared)

Putative Sly Promoter GTCCACATTATTAAATGTTTATGCACATAAATAAATAAAAAACCAAAAATGCTTACAAGA 239

Putative Orly Promoter GTCCACATTATTAAATGTTTATGCACATAAATAAATAAAAAACCAAAAATGCGTACAAGA 240

**************************************************** *******

<------ CdxA (shared)

<------ CdxA (shared)

------> CdxA (shared)

<------ CdxA (shared)

<------ CdxA (shared)

<------ CdxA (shared)

<----------- HFH-2 (shared)

<----------- HNF-3b (shared)

--------> Pbx-1 (shared)

--------> Pbx-1 (shared)

-------------> Oct-1 (shared)

<------------- Oct-1 (shared)

---------------------> Oct-1 (shared)

------> SRY (shared)

------------> XFD-1 (shared)

Putative Sly Promoter GCCTCTACAGCTGAAATTAAGTAGAGCCATTTGCTGTATTTCATATGTTACATATTTGTT 299

Putative Orly Promoter GCCTCTACAGTTGAAATTAAGTAGAGCCATTTGCTGTATTTCATATGTTTCATATTTGTT 300

********** ************************************** **********

<------ CdxA (shared)

------------> E4BP4 (*Sly* only)

----------- HNF-3b (shared)

----------> HLF (*Sly* only)

<------- Nkx-2.5 (shared)

------> Nkx-2.5 (shared)

<------ Sry (*Orly* only)

----------> VBP (*Sly* only)

Putative Sly Promoter CTGGATTTTCAAGTTTGTAAGCACTTGTCAACCAACAAACTGGAAATCATTTGTCTTAAA 359

Putative Orly Promoter CTGGATTTTCAAGTTTGTAAGCAATTATCAACCAACAAACTGGAAATCATTTGCCTTAAA 360

*********************** ** ************************** ******

-------🡪 c-Ets-1 (shared)

-----------> c-Myc/Max (*Sly* only)

------> CdxA (*Orly* only)

<----- CdxA (shared)

--------------> GATA-1 (*Orly* only)

> HNF-3b (shared)

------> Nkx-2.5 (shared)

<------- Nkx-2.5 (shared)

--------> Pbx-1 (*Orly* only)

---------------> S8 (*Orly* only)

Putative Sly Promoter GCCAGTAGATTACTCCCACCTTCTAAAATCTCCCTTCAAAGT------ACTTGGTATTCC 413

Putative Orly Promoter GCCAGTGGCTTATTCCCACCTTCTAAAATCTCCCTTCAAAGTGTTGGTACTTGGTATTCC 420

****** * *** ***************************** ************

- CdxA (shared)

----------> deltaE (shared)

<----------- Ik-2 (*Orly* only)

<------- Ik-3 (shared)

<--- STATx (*Sly* only)

Putative Sly Promoter GTGAACGCATGCGCAGGGTGTATTCAGCCAATCAGCACAGTCCTTTGGTGAGACCTATTT 473

Putative Orly Promoter ATGAACGCATGTGCAGGGGGTATTTAGCCAATCAGCACAGTCCTTTGGTGAGACCTGTTT 480

********** ****** ***** ******************************* ***

------------> C/EBP (shared)

<--------- GATA-1 (shared)

-------- HNF-3b (shared)

----- Ik-3 (shared)

<------------- NF-Y (shared)

----> STATx (*Sly* only)

Putative Sly Promoter GCTCCAGCTGGCATCACAAAGGATCCTCTGAGGCTTCTGTCTGGGTGTGGCCCCTGACAA 533

Putative Orly Promoter GCTCCAGCTGGCATCACAAAGGATCCTCTGAGGCTTCTGTCCGGGTCTGGCTCCTGACAA 540

***************************************** **** **** ********

<------ CdxA (shared)

---> HNF-3b (shared)

----- v-Myb (*Sly* only)

Putative Sly Promoter CGGTTTTTTTTTTTTTTTTTTTTTTTTTTTTTTTTTTTTTTTTTTTTTTGCCATTGAGGA 593

Putative Orly Promoter C-----TTTTTTTTTTTTTTTTTTTTTTTTTTTTTTTTTTTTTTTTTTTGCCATTGAGGA 595

* ******************************************************

< C/EBP (shared)

-----------> HFH-2 (*Sly* only)

---> v-Myb (*Sly* only)

Putative Sly Promoter GCTAAGC**ACAGAAGGAT** 610

Putative Orly Promoter GCTAAGC**ACAGAAGGGT** 612

*************** *

------------ C/EBP (shared)

**BOLD TEXT** indicates the transcription start site of *Sly* and corresponding location in *Orly*. Transcription factor binding sites predicted by TFSEARCH.
