# Supplementary material for: Bidirectional transcription of a novel chimeric gene mapping to mouse chromosome Yq
Source: BMC Evol Biol. 2007 Sep 24;7:171. doi: 10.1186/1471-2148-7-171 (PMC2212661; doi:10.1186/1471-2148-7-171)
Supplement: Additional file 3 — ClustalW alignment of Ssty1 and Ssty2. Annotated output from the ClustalW programme, aligning Ssty1 and Ssty2. Exons, coding region and the polyadenylation signal are highlighted for both genes. [file 1471-2148-7-171-S3.doc]

CLUSTAL W (1.83) MULTIPLE SEQUENCE ALIGNMENT

SSTY1_GENOMIC_REFERENCE TGCCTAGAAGGTGTGTCCACTATTTTCCCAGTGGTCTGTGAAAGGCAAGC 50

SSTY2_GENOMIC_REFERENCE TGAGTTCACTATCTTCACAGTGGTGTGTCAAAGCCAGGT 39

** ** ****** *** ******* *** **** ** *

SSTY1_GENOMIC_REFERENCE CAGCTCCTGAACTCCAACTTGTTCCTCAAGCTTTTGTGGCCTGGTCTGTA 100

SSTY2_GENOMIC_REFERENCE CATTTTTT--------------CCCACAGGCTTTTGGTGCCTGGTCTGTA 75

** * * ** ** ******* ************

SSTY1_GENOMIC_REFERENCE TCAAAAGACAGAATCTTGACGACTAAGACATTTTCTT-TGGGTAAGTACA 149

SSTY2_GENOMIC_REFERENCE TCAGGAGA-----TCTTGACGACTAGGATATTTTTCTATGGGTAAGTACA 120

*** *** ************ ** ***** * ************

SSTY1_GENOMIC_REFERENCE TTTATGAAGTAACTGGGACCATCATAGCTATAGAAAGCTGAAAGTTTTCC 199

SSTY2_GENOMIC_REFERENCE TTTATGAAGTAATTGGGACCCTCATAGCTACAGAAAGCTAAAAATTTTCT 170

************ ******* ********* ******** *** *****

SSTY1_GENOMIC_REFERENCE CTCCCACACTGAGTAACTGTACAGTCTAAATTCTCCCATGTTTGG-GGCC 248

SSTY2_GENOMIC_REFERENCE CTCCTACAGTGAGTTACTGTACATTCTACTTTC-CCCATGTTTATTAGCA 219

**** *** ***** ******** **** *** ********* **

SSTY1_GENOMIC_REFERENCE AGGGGCATGGTTGATCCAGTTTAGTTGATCTCCTGAATTTATTATCCTTG 298

SSTY2_GENOMIC_REFERENCE AGGGAGATGAATGGTCTAGGTTAGTTTATCTCCTGAATTTATTATCCTTG 269

**** *** ** ** ** ****** ***********************

SSTY1_GENOMIC_REFERENCE CTAACGATATCTCCTTCAAAATTCCATTAAAATGCCAAAGCTCTTCATTG 348

SSTY2_GENOMIC_REFERENCE CTAACCATATCTAGATCACAATTCCACTAAAATGCCAAAGCTCCTGTTTG 319

***** ****** *** ******* **************** * ***

SSTY1_GENOMIC_REFERENCE TCCTTATGTATTTTAGAAGATGTAACTTCTAAATGGCTGATATTGCCTGG 398

SSTY2_GENOMIC_REFERENCE TCCATGTGCATGTTAGAAGTTATTAGTTCTAAAAGGCTGATATTGCCTCG 369

*** * ** ** ******* * * * ******* ************** *

SSTY1_GENOMIC_REFERENCE ACTTGTTGAGG----CCGGAGTTGCAGTGGCA-AATAA--TCACCAAATA 441

SSTY2_GENOMIC_REFERENCE ACTTTTTGAGTTGTTCTTGAGTT-CAGTGATGCAATGGCTTCATCGAATA 418

**** ***** * ***** ***** *** *** * ****

SSTY1_GENOMIC_REFERENCE ATCCTGAGGCTGGGTCAGGTAACACAATACAGAAGGCCAGTGGTGTGCTG 491

SSTY2_GENOMIC_REFERENCE ATTCTGAAGCTGGGTCAGGAGAAACAATAAAGAAGGACAGTGGTGTGCTG 468

** **** *********** * ****** ****** *************

SSTY1_GENOMIC_REFERENCE TGTCCCTGGGA---TATTTGTGAATATTGCCACCTATTTCACAGCTTGCA 538

SSTY2_GENOMIC_REFERENCE TGTCCCTGGGAAGATATTTGTGAATGCAAGAACTTAGCTCAGAGCTTACG 518

*********** *********** ** ** *** ***** *

SSTY1_GENOMIC_REFERENCE GGTACAAGAGAGGGTAGGTGACAACTCTGGCTCATTTTCTTTATTCTGTT 588

SSTY2_GENOMIC_REFERENCE CGTACAAGAGAATCATGGTGACCACTCTGGCC--TTTTTTTTTTTTTCTC 566

********** ****** ******** **** *** ** * *

SSTY1_GENOMIC_REFERENCE CTCTCCTTGATCATTTTGGGTAAAAGGCATACCAACTTCCACATTGACAT 638

SSTY2_GENOMIC_REFERENCE TTCTTCTTGATCATTGGGGGTAATTGTCATAGCACCACCTAAATTGACAT 616

*** ********** ****** * **** ** * * * ********

SSTY1_GENOMIC_REFERENCE AGACATGCAAGGAGATGTTCCTCAGCAGAGGCAAATTAGCTCAGGGCTGA 688

SSTY2_GENOMIC_REFERENCE AGAAATTCAAGGAGAGGATCCTCATCAGAGACAAATTTACTCAGGTCTGA 666

*** ** ******** * ****** ***** ****** ****** ****

SSTY1_GENOMIC_REFERENCE AGACATA----TTCATTTGAGTATATTATTCTGAGCTCTCGAGAATTACT 734

SSTY2_GENOMIC_REFERENCE AGAAATAAATGTTGATCTGAGTATGTTATTCTGAGCTCTAGAGAATTACT 716

*** *** ** ** ******* ************** **********

SSTY1_GENOMIC_REFERENCE TGTTAATTTCCCTTTGATAATTTCCTTGTTTCCTTTATTTATTTGTTTGT 784

SSTY2_GENOMIC_REFERENCE TGTTAATTACACTATTTTTATTTCCTTGTTTCCCTTGTTTCTTTGTTT-- 764

******** * ** * * ************** ** *** *******

SSTY1_GENOMIC_REFERENCE TGTTGTTGTTGTTGTTTTGTTTGTTTGTTTTTTCTTTGGATGGGTGTTTA 834

SSTY2_GENOMIC_REFERENCE --------------CTTTGTTTGTTTGTTTGCTTGCT-------TGGTTA 793

*************** * * ** ***

SSTY1_GENOMIC_REFERENCE ATTTTACTTTGAGTGTGTTTGTGTGTAGCTTTGGCTGTTAGGAAAATCAC 884

SSTY2_GENOMIC_REFERENCE ATTTTA-TTTGAGTTTCTGTATGTTTAGATTTGGCTGTCAGGAAACTCAC 842

****** ******* * * * *** *** ********* ****** ****

SSTY1_GENOMIC_REFERENCE TTTGTAGAATAGACTAGCCTTTATCTCACTAAGGTTTGCCTCCCTCTGCC 934

SSTY2_GENOMIC_REFERENCE TGTGTAGACCAGACTAGTCTTTCTCTCACTAAGGTCTGCCTCACTCTGCC 892

* ****** ******* **** ************ ****** *******

SSTY1_GENOMIC_REFERENCE TCTTGAGTACTAGGATTAAAGATTTGGGTTACTACCATCTCCTATTAACT 984

SSTY2_GENOMIC_REFERENCE TCTTGAGTACTATGATTAAAGTTATGGGTCACTACCACTTCCTATTAACT 942

************ ******** * ***** ******* ***********

SSTY1_GENOMIC_REFERENCE TCTG-ATTTGATTGTTTGTTTTGTTTTGTTTTGTTTGTTTTTCTTTTTTG 1033

SSTY2_GENOMIC_REFERENCE TCTCCATTTGATTGTTTGTTTTGTTATTTTTTGTTTGAATTTCTCTTTTG 992

*** ******************** * ********* ***** *****

SSTY1_GENOMIC_REFERENCE TTTTTTGTTTGTATGTTTTGTTTTGTTTTTGTTGGTTTTTTGTT-TTAGT 1082

SSTY2_GENOMIC_REFERENCE TTTGTT---------TTTCGTTTGATTTTCGTTTGTTTTCTTTTACTTGA 1033

*** ** *** **** **** *** ***** * ** * *

SSTY1_GENOMIC_REFERENCE TGTTAATGTTGTAGCTGATTTGGAGACATTTTTTCTTTGTCTATCACTGG 1132

SSTY2_GENOMIC_REFERENCE TGTTGTTGTTGTTGTTGTTGTTGAGACAGGGTTCCTCTCTATAGCACTGA 1083

**** ****** * ** * * ****** ** ** * * ** *****

SSTY1_GENOMIC_REFERENCE CTGCCCTGGAACTCACTTTGTAGACCAGGCTGGTCTCTAAGTCAGAAATC 1182

SSTY2_GENOMIC_REFERENCE CTGCCCTGGAACTCACTTTGTAGACCAGGCTGGTCTCTAATTTAGAAATC 1133

**************************************** * *******

SSTY1_GENOMIC_REFERENCE TGCCTGACCCTGCCTTCTGAAAGCTGGGATTAAATGTGTGTGCCACTATA 1232

SSTY2_GENOMIC_REFERENCE TGCCTGTCCTTGCCTTCTGAGTTCTGGGATTAAATGCGTGTACCACCATG 1183

****** ** ********** ************* **** **** **

SSTY1_GENOMIC_REFERENCE GCTGGATTATGAGCTTCTTATTTTGATTAAATTAAGTTTAGAAGCCCAAA 1282

SSTY2_GENOMIC_REFERENCE GCTGACTTATTAACTTCTTATTTTTATAAAATTAATTTTAGGAGCTCAAA 1233

**** **** * *********** ** ******* ***** *** ****

SSTY1_GENOMIC_REFERENCE CATGTTTAGCATAACCTTCTTGACCTGCTCAAAAATTCCATTAGAGAAGA 1332

SSTY2_GENOMIC_REFERENCE TCTGTTTAGCATAATCTTCTTCACCTGCTCAGAAGTTCTCGTAGAGAAGA 1283

************ ****** ********* ** *** *********

SSTY1_GENOMIC_REFERENCE GCCTTGGTCCTGCATTCTCTCCCACCCTACACCCATTGCCTTTCAGTAAC 1382

SSTY2_GENOMIC_REFERENCE GTCTTGGTCCAGCATTCACTCTCAACCCATAACCATTGCCCATCAGGAAT 1333

* ******** ****** *** ** ** * * ******** **** **

SSTY1_GENOMIC_REFERENCE TCATATATATGACAGTAGCAACAGCATTGTGTCTGGTTTAAACAGGCAAT 1432

SSTY2_GENOMIC_REFERENCE TCATATATATGACAGAGGCAACAGCATTGTGTCTGGTTTAAACGGGCAAG 1383

*************** ************************** *****

SSTY1_GENOMIC_REFERENCE ATCTTCAATCCAAGGCAATGGGCAAGTAT-ATTTGAACACAGAATGATAG 1481

SSTY2_GENOMIC_REFERENCE ATCTTCAGTCCCAGGCAATGAGCAAGTATGATATTTGAAGGGAATGGAAG 1433

******* *** ******** ******** ** * * ***** **

SSTY1_GENOMIC_REFERENCE CCACAAGACATCTGCCTGGGTTTGGTGTGGTATACCACAGCAAGGCTACA 1531

SSTY2_GENOMIC_REFERENCE CCACAAGACA--------------GTGTGATCTACCACAACAGGTCTACA 1469

********** ***** * ******* ** * *****

SSTY1_GENOMIC_REFERENCE GCCATGTAGAAGAAAACACCTTTGAAGAGGCTTAGCATGTCATCCCTCAT 1581

SSTY2_GENOMIC_REFERENCE GCCAGGTAGAAGACAATACATCTGAAGAGTCTGAGCATGACATCACTCAA 1519

**** ******** ** ** * ******* ** ****** **** ****

SSTY1_GENOMIC_REFERENCE GAAGAAGAGGAGGAGGAAGTCTTCTTCCAACACCCTGAGGAATATTGTCA 1631

SSTY2_GENOMIC_REFERENCE GAAGAAGAGTAGGAGGAAGCCTTCTTCCCAGGCCCTGGGGAATATTGTTG 1569

********* ********* ******** * ***** **********

SSTY1_GENOMIC_REFERENCE GCTGCAGAATTTCTCACAGTTGGAAGGAAGGTAATGAGCCTGTCACCCAA 1681

SSTY2_GENOMIC_REFERENCE GCTGCAGAATTTCTCACGGGTGGAAGGAAGGTAATGAGCCTGTCACCCAT 1619

***************** * *****************************

SSTY1_GENOMIC_REFERENCE TGGAAGGCCATAGTTCTAGATCAACTGCCAACAAACCCTTCTCTTTACTT 1731

SSTY2_GENOMIC_REFERENCE TGGAAGGCCATCATTCTAGGTCAACTGCCAACAAACCCTTCTCTTTATTT 1669

*********** ****** *************************** **

SSTY1_GENOMIC_REFERENCE GGTGAAGTATGATGGAATTGACAGCATCTACGTACTGGAGCTCTACAGTG 1781

SSTY2_GENOMIC_REFERENCE GGTGAAGTATGACGGAATTGACAGTGTCTACGGACAGGAGCTCCACAGCG 1719

************ *********** ****** ** ******* **** *

SSTY1_GENOMIC_REFERENCE ATGACAGGATTTTAAACCTTAAGGTTTTGCCTCCCATAGTAGTATTTCCT 1831

SSTY2_GENOMIC_REFERENCE ATGAGAGGATTTTAAATCTTAAGGTCTTGCCTCACAAAGTAGATTTTCCT 1769

**** *********** ******** ******* ** ***** ******

SSTY1_GENOMIC_REFERENCE CAGGTGAGGGATGCCCACCTCGCCAGAGCCCTGGTTGGCAGAGCGGTACA 1881

SSTY2_GENOMIC_REFERENCE CAGGTGAGGGAGGTCCACCTCGCAGGCACACTGGTTGGCAGAGAGGTACA 1819

*********** * ********* * * ************* ******

SSTY1_GENOMIC_REFERENCE ACACAAATTTGAGAGGAAAGATGGCTCTGAGGTCAACTGGAGGGGGGTGG 1931

SSTY2_GENOMIC_REFERENCE ACACAAATTTGAGGGGAAAGATGGCTCTGAGGACAACTGGAGTGGGATGG 1869

************* ****************** ********* *** ***

SSTY1_GENOMIC_REFERENCE TGCTAGCCCAGGTGCCAATCATGAAGGATTTGTTTTACATTACCTACAAG 1981

SSTY2_GENOMIC_REFERENCE TGCTAGCCCAGGTGCCATTCTTACAGGACTATTTTTACATTTCCTACAAG 1919

***************** ** * **** * ********* ********

SSTY1_GENOMIC_REFERENCE AAGGATCCAGCTCTCTATGCTTATCAGCTCCTGGATGACTACAAGGAAGG 2031

SSTY2_GENOMIC_REFERENCE AAGGATCCGGTCCTCTACGTCTATCAGCTCCTGGATGACTACAAGGAAGG 1969

******** * ***** * *****************************

SSTY1_GENOMIC_REFERENCE TAACCTCCACATGATTCCAGACACTCCTCCGGCTGAGGAGAGATCAGGAG 2081

SSTY2_GENOMIC_REFERENCE TAACCTCCACATCATTCCAGAGACCCCTCTGGCTGAGGCGAGATCAGGTG 2019

************ ******** ** **** ******** ********* *

SSTY1_GENOMIC_REFERENCE ATGACAGTGATGTGTTGATTGGTAACTGGGTGGAGTACACCAGAAAAGAT 2131

SSTY2_GENOMIC_REFERENCE ATGACAATGACTTCTTAATAGGTTCCTGGGTGCAGTACACCAGAGATGAT 2069

****** *** * ** ** *** ******* *********** * ***

SSTY1_GENOMIC_REFERENCE GGTTCCAAAAAGTTCGGAAAGGTTGTTTACCAAGTTCTAGCCAATCCTTC 2181

SSTY2_GENOMIC_REFERENCE GGATCCAAAAAGTTCGGAAAGGTTGTTTACAAAGTTCTAGCCAATCCTAC 2119

** *************************** ***************** *

SSTY1_GENOMIC_REFERENCE CGTGTACTTTATCAAGTTTCATGGTGACATCCATATCTATGTCTATACTA 2231

SSTY2_GENOMIC_REFERENCE TGTGTACTTTATCAAATTTCTCGGTGACCTCCATATCTATGTCTATACTC 2169

************** **** ****** ********************

SSTY1_GENOMIC_REFERENCE TGGTGCCAAAGATTCTTGAAGTTGAAAAA--TCATAAAGTACAGAAACGT 2279

SSTY2_GENOMIC_REFERENCE TGGTGTCAAATATCACTTAAATTGAAAAAAATCACAAAGTACAGAAATGT 2219

***** **** ** * ** ******** *** ************ **

SSTY1_GENOMIC_REFERENCE AAACATATAGGACTGGAAGAAAAAAAAAGTTTTTTTTTTCCTGTGTTGGC 2329

SSTY2_GENOMIC_REFERENCE AAACTTATAGGATTG-----AAAAAAAAATGTTTGTTTTCCTGTGTTGGG 2264

**** ******* ** ******** * *** **************

SSTY1_GENOMIC_REFERENCE TACATAAGGGTCTTTGATAATCCCAGTATCTTTGCCAATAAAATGTGTTT 2379

SSTY2_GENOMIC_REFERENCE TACCTATGGGTCTTTGACAACCTCAGTATCTTTGTCAATAAAATTTGTTT 2314

*** ** ********** ** * *********** ********* *****

SSTY1_GENOMIC_REFERENCE TGTTCT 2385

SSTY2_GENOMIC_REFERENCE TGTTCTAAAAATTAA 2329

******

INTRON

EXON

CODING REGION

POLYADENYLATION SIGNAL
