# Supplementary material for: Bidirectional transcription of a novel chimeric gene mapping to mouse chromosome Yq
Source: BMC Evol Biol. 2007 Sep 24;7:171. doi: 10.1186/1471-2148-7-171 (PMC2212661; doi:10.1186/1471-2148-7-171)
Supplement: Additional File 5 — UPGMA Phylogenetic tree of Ssty1, Ssty2 and Orly. Phylogenetic tree of Ssty1, Ssty2 and Orly gene copies using the same alignment used for Figure 12. The UPGMA algorithm was used, rather than the neighbour-joining algorithm used for Figure 12. [file 1471-2148-7-171-S5.ppt]

## Slide 1
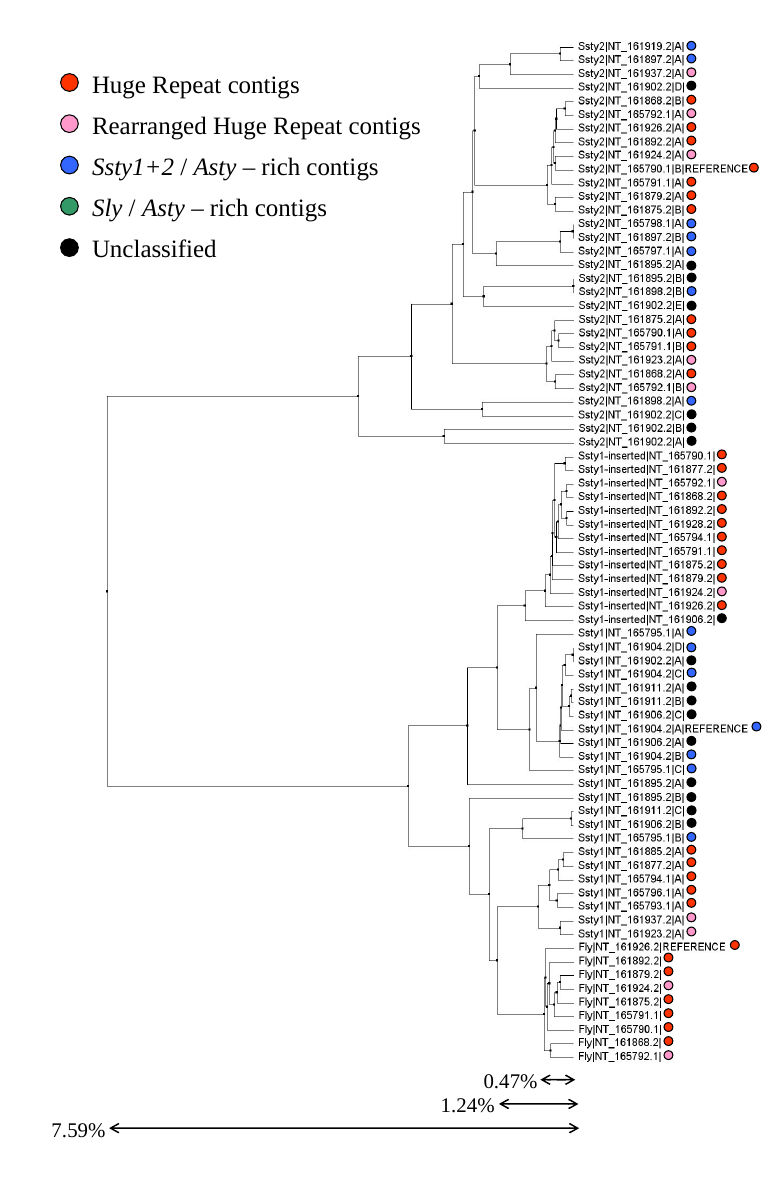

Huge Repeat contigs
Rearranged Huge Repeat contigs
Ssty1+2 / Asty – rich contigs
Sly / Asty – rich contigs
Unclassified
0.47%
1.24%
7.59%
