# Supplementary material for: Bidirectional transcription of a novel chimeric gene mapping to mouse chromosome Yq
Source: BMC Evol Biol. 2007 Sep 24;7:171. doi: 10.1186/1471-2148-7-171 (PMC2212661; doi:10.1186/1471-2148-7-171)
Supplement: Additional File 6 — UPGMA Phylogenetic tree of Asty and Orly. Phylogenetic tree of Asty and Orly gene copies using the same alignment used for Figure 13. The reference Astx sequence was used as the outgroup to root the tree. The UPGMA algorithm was used, rather than the neighbour-joining algorithm used for Figure 13. [file 1471-2148-7-171-S6.ppt]

## Slide 1
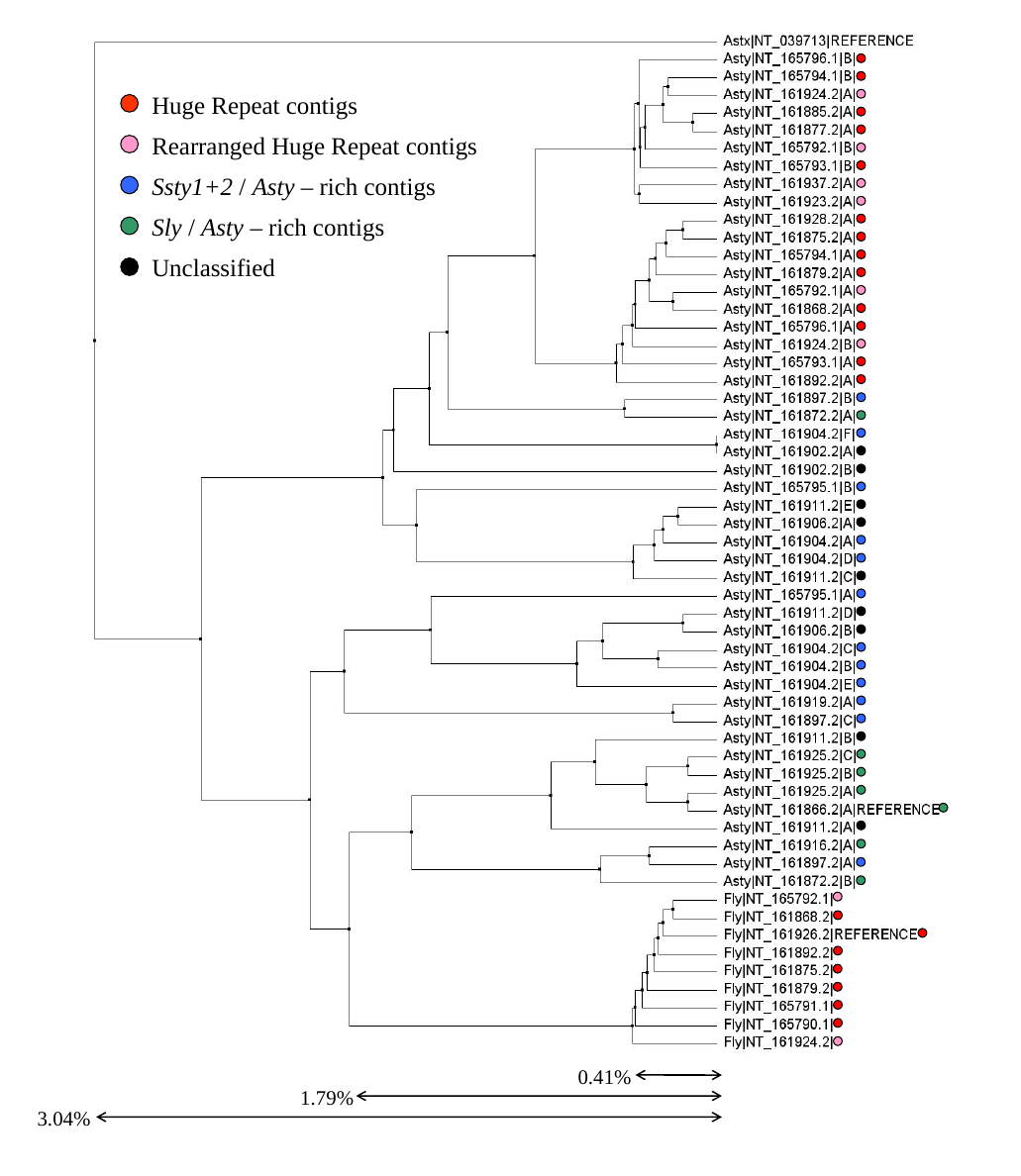

Huge Repeat contigs
Rearranged Huge Repeat contigs
Ssty1+2 / Asty – rich contigs
Sly / Asty – rich contigs
Unclassified
0.41%
1.79%
3.04%
